# Recent Advances on Neuromorphic Systems Using Phase-Change Materials

**DOI:** 10.1186/s11671-017-2114-9

**Published:** 2017-05-11

**Authors:** Lei Wang, Shu-Ren Lu, Jing Wen

**Affiliations:** 1 School of Information Engineering, Nanchang HangKong University, Nanchang, 330063 People’s Republic of China; 2 Department of Automatic Control, School of Information Engineering, Nanchang Hangkong University, Nanchang, 330069 Jiangxi People’s Republic of China

**Keywords:** Phase-change materials, Neuromorphic, Neuron, Synapse, STDP, Brain

## Abstract

Realization of brain-like computer has always been human’s ultimate dream. Today, the possibility of having this dream come true has been significantly boosted due to the advent of several emerging non-volatile memory devices. Within these innovative technologies, phase-change memory device has been commonly regarded as the most promising candidate to imitate the biological brain, owing to its excellent scalability, fast switching speed, and low energy consumption. In this context, a detailed review concerning the physical principles of the neuromorphic circuit using phase-change materials as well as a comprehensive introduction of the currently available phase-change neuromorphic prototypes becomes imperative for scientists to continuously progress the technology of artificial neural networks. In this paper, we first present the biological mechanism of human brain, followed by a brief discussion about physical properties of phase-change materials that recently receive a widespread application on non-volatile memory field. We then survey recent research on different types of neuromorphic circuits using phase-change materials in terms of their respective geometrical architecture and physical schemes to reproduce the biological events of human brain, in particular for spike-time-dependent plasticity. The relevant virtues and limitations of these devices are also evaluated. Finally, the future prospect of the neuromorphic circuit based on phase-change technologies is envisioned.

## Review

### Background

**Fig. 1:**
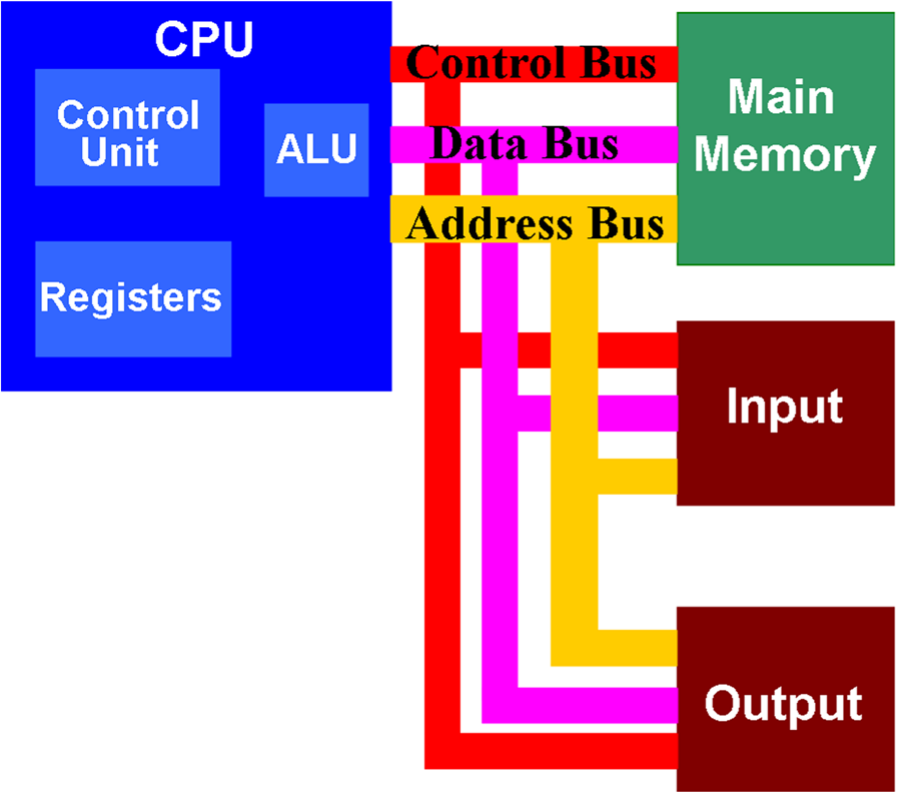
von Neumann architecture of modern computer

Today, digital computer, commonly considered as a milestone in the history of human life, has a pervasive influence on every citizen’s daily activities involving business, education, entertainment, and sports. As a physical device while manipulated by the operational system, the prosperity of the digital computer aggressively lies on the progress of both hardware and software technologies. Recent technological developments on ultra-large-scale integration (ULSI) allow millions or even billions of electronic components to be integrated on a single semiconductor chip, significantly improving the physical performances of the modern computer. Under this circumstance, it is not too naïve to imagine that human being will be govern by computer machines one day that has been frequently described in scientific fictions, particularly after the recent victory of ‘AlphaGo’ over the top human Go player [1]. However, thanks to the architectural difference between computer and human brain, it is not possible for digital computer to outperform the biological brain in the near future. It is well known that modern computer usually makes use of the so-called von Neumann architecture that consists of three main components [2], i.e. processor, main memory, and bus, as shown in Fig. 1. The processor, also known as central processing unit (CPU), comprises arithmetic logic unit (ALU), control unit, and register. As implied by their names, ALU is responsible for all the arithmetic and logical operations such as addition, subtraction, AND, and OR functions, while the control unit decodes the instructions and controls all other internal components of the CPU. The register is mainly used to store the data during execution. After all the essential computations, the processed data is sent back from the CPU to the main memory where the data is stored through the data bus, whereas address bus and control bus are employed to determine the address of the data inside the memory and the type of the operations (e.g. write and read) between CPU and memory, respectively.

According to above descriptions, an apparent feature of modern computer adopting von Neumann architecture is that CPU where data is processed is separated from the main memory where data is stored by bus. As a consequence, CPU needs to retrieve data from the main memory for any necessary processing, after which data is transferred back to the main memory for storage. The fact that bandwidth between CPU and the main memory (also called data transfer rate) is much lower than the speed that a typical CPU can work severely limits the processing speed of the modern computer, which is known as von Neumann bottleneck. In order to circumvent the von Neumann bottleneck, several advanced technologies such as Cache memory, multi-threading core, and low-latency command channel have been proposed in the past to increase the processing speed of the modern computer. These approaches seem to be viable for the cases with less repeated operations that only cope with relatively small amount of the processing data, while failing to satisfy the demands from data-centric applications that usually require often-repeated transient operations on a vast amount of the digital data such as real-time image recognition and natural language processing [3]. Therefore, the current consensus is that it is inevitable to have an unprecedented revolution from von Neumann architecture to non-von Neumann architecture so as to utterly eliminate the von Neumann bottleneck.

**Fig. 2:**
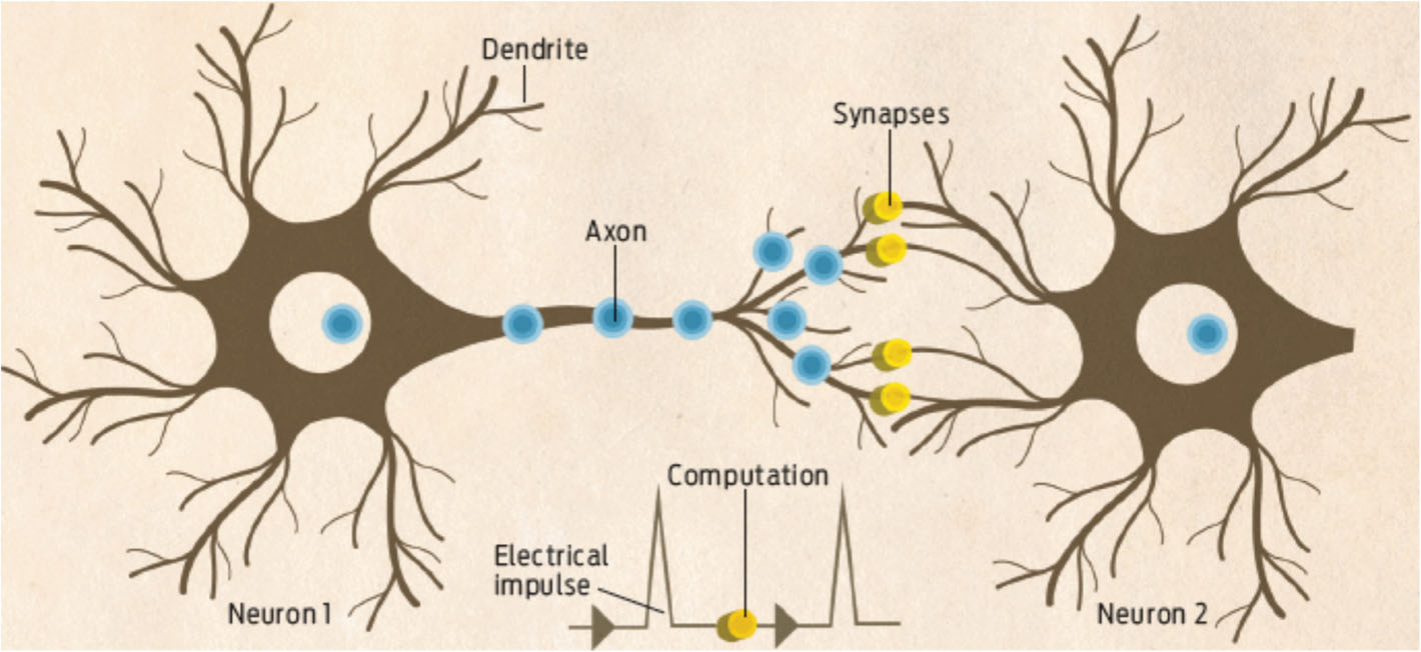
Neuron structure in the human brain. Reprinted with permission from [4]

Fortunately, an ideal computing system that adopts non-von Neumann architecture has been existing for millions of years, which is the human brain. The human brain comprises many types of cells within which the core component is called neuron [4], as shown in Fig. 2. The highly importance of the neuron for human brain stems form its ability to process and transmit information through electrical and chemical signals. According to Fig. 2, a neuron is made up of a cell body (also called soma), dendrites, and an axon. Information usually in the form of an electrical or chemical signal is transferred from an axon of one neuron towards the conjunction of its axon and dendrites of the neighbouring neurons, also known as synapse. The synapse can evaluate the importance of the received information by integrating it with the strength of the synapse (synaptic weight), and subsequently distribute information to even more neurons through their respective axons. Differing from the digital computer, human brain performs the information processing during the transferring period, and there is only a single value by the time that information reaches the neighbouring neurons. This clearly indicates an encouraging finding that human brain allows information storage and processing to occur at the same time in the same place. Due to this attractive feature, human brain whose neural networks consists of ~10^11^ neurons and ~10^15^ synapses enables an operation frequency of 1–10 Hz on the power budget of 10–100 W [5], corresponding to an energy consumption of 1–10 fJ per synaptic event [5].

Thanks to the exceptional capability of the human brain, it is natural to conceive the possibility of building a super-intelligent computer that reproduces the neural networks of the human brain to completely overcome the von Neumann architecture, leading to the prosperity of artificial intelligence (AI). One possible way to achieve brain-like computer is to simulate the behaviours and connections between biological neurons inside the human brain using conventional computers or even so-called supercomputers, replying on the recent progress of the software algorithms. In spite of its advantageous flexibility and availability [6, 7], the software-based approaches fail to cope with large-scale tasks such as pattern recognition, learning, and intelligent cognition [8, 9], and also causes several orders of magnitude higher energy consumption than the human brain [10]. In this case, vast majority of research efforts has been recently devoted to exploiting a novel hardware architecture that can emulate both the biological structure and the biological function of the human brain, delivering the debut of neuromorphic engineering that can be dated back to 1980s [8]. However, the concept of neuromorphic has not received considerable attentions until the presence of the emerging non-volatile memories (NVM) such as Ferroelectric random access memory (FeRAM) [11, 12], magnetic random access memory (MRAM) [13, 14], phase-change random access memory (PCRAM) [15, 16], and resistive random access memory (ReRAM) [17, 18], as well as the physical realization of the early proposed ‘memristor’ concept [19–21]. These innovative devices are capable of storing information on an ultra-small region during ultra-short interval with ultra-low energy consumption, closely resembling the attractive features of the human brain. Perhaps most enticingly, some physical properties of these devices that depend on their previous states can be successively tailored by the external stimulus and be stored even after power-off, very analogous to the behaviours of the biological synapse. As a result, several nanoscale electronic devices that include metal oxides [22, 23], solid-electrolytes [24, 25], carbon nanotubes [26, 27], organic electronics [28, 29], spin transfer torque MRAM [30, 31], and phase-change memory (PCM) [32] based on the aforementioned technologies have been proposed to systematically mimic the biological function of the human brain, in particular the learning and memorization capabilities govern by the synapse. It should be noticed that due to the extremely complexity of the human brain, none of these available electronic devices has exhibited the promising potential to prevail or even meet the physical performances of the biological synapse to date. However, the nanoscale electronic synapse based on PCM technology seems to be more promising than its compatriots, as phase-change materials has already gained widespread applications from the conventional optical disc [33] to the recently emerging all-photonic memory [34], and PCRAM is considered as the leading candidate for the so-called universal memory for NVM applications [35, 36]. More importantly, a wealth of theoretical knowledge and practical experiences that concerned the electrical, optical, thermal, and mechanical properties of the phase-change materials has been accumulated along with the development of the PCMs that has been under intensive study for the last two decades. For above reasons, a comprehensive review about physical principles and recent developments of the nanoscale electronic synapse using phase-change devices becomes indispensable in order to help researchers or even ordinary readers to deeply understand the physical way that the phase-change electronic synapse can imitate the biological synapse, and thus stimulate more research efforts into the establishment of the design criterion and performance requirement for the future artificial synapse device to ultimately compete with the human brain.

Here, we first introduce the fundamental biological behaviours of brain neurons and synapses that are the prerequisites for designing appropriate artificial synapses, followed by a brief overview of the phase-change materials and its applications on NVM applications. Then, we present different types of nanoscale electronic synapse using phase-change devices as well as their respective advantages and disadvantages for neuromorphic applications. The future prospect of the artificial synapse using phase-change materials is also discussed.

### Biological Behaviours of the Human Brain

**Fig. 3:**
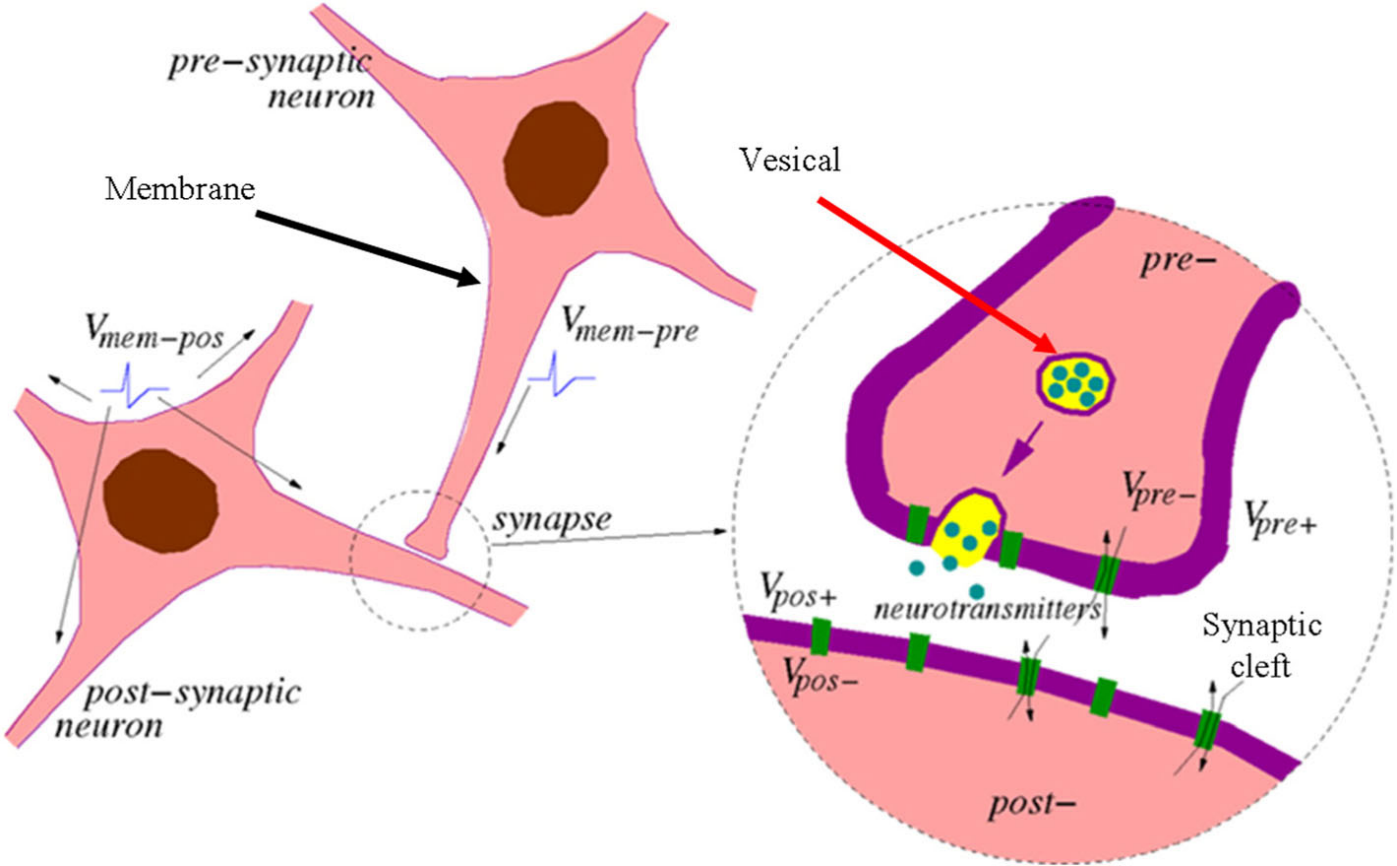
Structure of the neuron and synapse and schematic of the synaptic junction between the presynaptic neuron and postsynaptic neuron. Reprinted with permission from [42]

Briefly speaking, the patterns of synaptic transmissions between different neurons inside the brain can be simply classified into electrical synapse and chemical synapse. Electrical synapse transmits signal directly by the drift of ions without involving any chemical reaction. In this case, electrical synapse usually exhibits a more rapid signal transmission that allows signal to flow bidirectionally between neighbouring neurons than chemical synapse and is frequently found on a nerve system that requires quick responses. However, electrical synapse is passive, which means that the signal received in the receiver side is the same as or smaller than the initial signal from the sender side, called ‘lack of gain’. Chemical synapses usually divided into excitatory synapse and inhibitory synapse allows signal to be transmitted unidirectionally between neuron sender (called presynaptic neuron) and neuron receiver (called postsynaptic neuron) through the release of neurotransmitter from presynaptic neuron and absorption of neurotransmitter from postsynaptic neuron [37]. The certain type of chemical synapse is completely determined by the receptors on the postsynaptic membrane. It has been reported that the gamma-aminobutyric acid (GABA) neurotransmitter is an inhibitory receptor [38] that leads to an inhibitory postsynaptic potential (IPSP) [39] and moves the postsynaptic neuron away from depolarization threshold, while glutamate neurotransmitter is an excitatory receptor [40] that exhibits an excitatory postsynaptic potential (EPSP) and drives postsynaptic neuron towards the depolarization threshold that can activate an action potential [41]. Various neurons can be connected to one certain neuron via their axon terminals and release neurotransmitters that stimulate excitatory and inhibitory receptors to yield EPSPs and IPSPs; subsequently, postsynaptic neuron integrates the EPSPs with the IPSPs to determine whether to excite or ‘fire’ an action potential or not. In this case, the resulting action potential is transferred to the synapse cleft through the axon, and merges the neurotransmitter vesicles with the presynaptic membrane at the axon terminal, thereby releasing the neurotransmitters into the synapse cleft. After diffusing through the synapse cleft, these neurotransmitters bind to and activate the receptor in the postsynaptic membrane, thereby changing the synapse weight. Such a chemical scheme is schematically shown in Fig. 3 [42]. As chemical synapse is known to be in charge of learning and memory, the ‘synapse’ mentioned in this review refers to chemical synapse, unless otherwise stated. A more detailed description of the biological behaviours of the chemical synapse is presented below from chemical point of view.

Note that neurons communicate with each other through so-called action potentials that can be interpreted as the transmitted electrical signals [43]. A liquid membrane separates neuron whose intracellular components are rich in K^+^ ions from outside environment whose extracellular component has high concentration of Na^+^ and Cl^−^ ions. This non-uniform ion distribution across the membrane polarizes the membrane with a certain resting potential between −90 and −40 mV [44], when the cell membrane does not experience any external stimulus, i.e. in its rest state. Nevertheless, when subject to an external electric stimuli above some certain threshold, the ions can exchange through voltage-gated ion channels that are activated by the change of the electrical membrane potential and ion pumps between intracellular and extracellular media to lower the chemical potential gradient [45–49]. Such a re-distribution of the ions across membrane leads to the depolarization of the neuron, also known as action potential firing responsible for the signal transmission from the neuron soma to the terminal of the neuron axon [43]. However, this depolarized state would soon restore to the previously rest state after Na^+^/K^+^ ion pumps recover its ion distribution to the rest state, called sodium-potassium adenosine triphosphatase (Na^+^/K^+^-ATPase) [50]. Therefore, the state change of the neuron or cell membrane is considered as ‘elastic’ meaning no permanent change occurring when the external stimuli exceeds the threshold value.

**Fig. 4:**
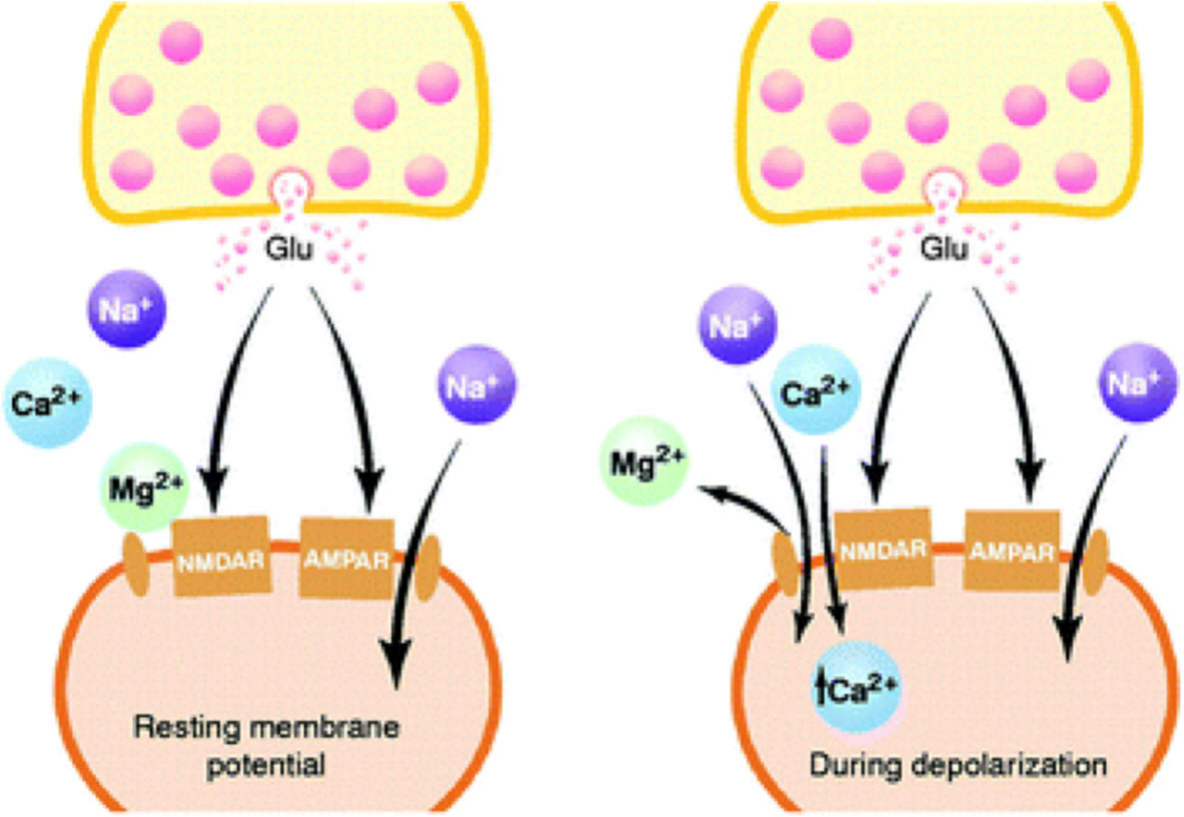
Potentiation procedures for a chemical synapse. The presynaptic activation releases glutamate neurotransmitters (Glu) that bind to NMDAR and AMPAR. Only the AMPAR opens the ion channels for monovalent cations (e.g. Na^+^) when the postsynaptic neuron is at rest state or polarized, while NMDAR opens the voltage-gated channels to allow Ca^2+^ to diffuse once the postsynaptic neuron is depolarized or activated. Reprinted with permission from [53]

Although the change of neuron states exhibit elasticity, the change of synapse weight however shows plasticity, simply meaning that the change can either last for a long time or a short time. Given its durability, a long-term activity-dependent synaptic plasticity has been widely believed to account for learning and memory [51]. The synaptic activity to induce a long-lasting increase on synapse weight is named as long-term potentiation (LTP) that is in charge of the long-term memory, whereas the synaptic activity to generate a long-lasting decrease on synapse weight is known as long-term depression. LTP is believed to be strongly related to the activities of the presynaptic and postsynaptic neurons. When the presynaptic neuron is activated, it releases various neurotransmitters within which glutamate neurotransmitter acts as a principle role in LTP [52]. The released glutamate is subsequently bound to *N*-methyl-d-aspartate receptor (NMDAR) that opens ion channels for both monovalent and divalent cations (Na^+^, K^+^, and Ca^2+^), and α-amino-3-hydroxy-5-methyl-4-isoxazolepropionic receptor (AMPAR) that opens ion channels for monovalent cations (Na^+^ and K^+^), which are located on the postsynaptic neuron [52]. Accordingly, the change of the electrical membrane potential causes the depolarization of the postsynaptic neuron that opens the voltage-gated ion channel of the NMDAR and consequently results in an increase of Ca^2+^ concentration that can be further enhanced through Ca^2+^ inward flux through L-type voltage-gated calcium channels (VGCCs) [53–57]. However, a weaker depolarization is unable to completely displace the Mg^2+^ ions that block NMDA ion channels and therefore prevents adequate Ca^2+^ ions from entering the postsynaptic neuron, yielding a lower intracellular Ca^2+^ concentration. This is considered as the induction of LTD. Figure 4 shows the schematics of the described process.

**Fig. 5:**
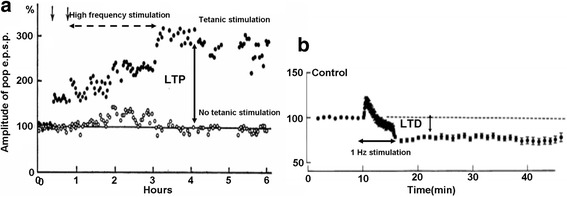
Experimental demonstration of LTP and LTD in a biological synapse showing synaptic conductance as a function of time. **a** The *solid circle* denotes the conductance arising from high-frequency tectonic stimulation which results in LTP, and the *open circle* indicates the conductance in the absence of tectonic stimulation. **b** The change in synaptic conductance stemming from low frequency stimulation, yielding LTD. The *dotted horizontal line* shows the conductance level without applying stimulation. Reprinted with permission from [5]

The mechanisms governing synaptic plasticity shown in Fig. 4 still remain mysterious. One hypothesis attributes the induction of synaptic plasticity to the relative rates between the pre- and postsynaptic action potentials also called spikes [58]. Based on rate-dominant speculation, high-frequency stimulations induce LTP, while low frequency stimulations bring about LTD, as illustrated in Fig. 5.

**Fig. 6:**
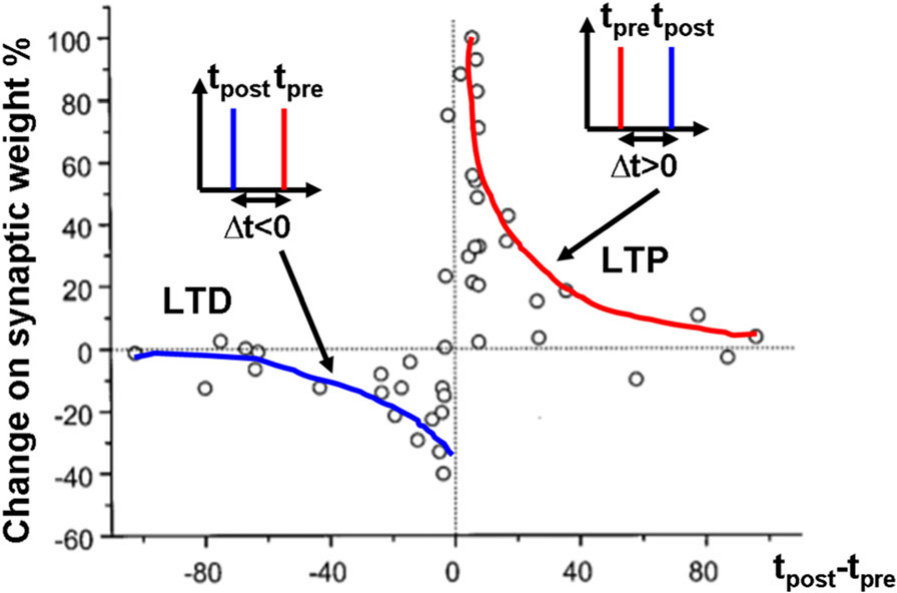
Experimental demonstration of percentage change on synapse weight as a function of the relative timing between pre- and post-synaptic spikes. LTP is generated when presynaptic spike precedes post-synaptic spike, whereas LID is induced when presynaptic spike lags post-synaptic spike. Reprinted with permission from [61]

An alternative to rate-dominant synaptic plasticity is time-dependent synaptic plasticity that ascribes the cause of long-term plasticity to temporal correlations between pre- and postsynaptic spikes [59]. The origin of this assumption can be dated back to 1949 when Donald Hebb proposed his famous Hebbian learning rule: ‘When an axon of cell A is near enough to excite cell B and repeatedly or persistently takes part in firing it, some growth process or metabolic changes take place in one or both cells such that A’s efficiency as one of the cells firing B, is increased’ [60]. Hebb suggested that the activations of pre- and postsynaptic neurons do not essentially induce the long-term plasticity, but the relative spiking time that is the time difference between presynaptic spike and postsynaptic spike plays the most important role on synaptic plasticity, usually referred to spike-timing-dependent plasticity (STDP). As illustrated in Fig. 6, the situation that presynaptic spike precedes postsynaptic spike results in LTP, while the synapse undergoes LTD when presynaptic spike lags postsynaptic spike [61].

**Fig. 7:**
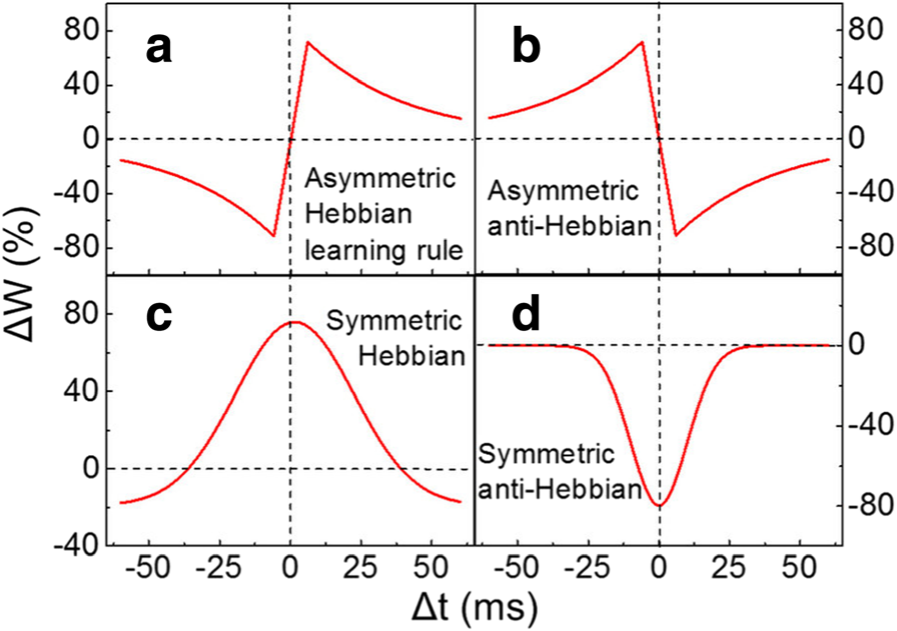
Ideal STDP learning functions. **a** Asymmetric Hebbian learning function. **b** Asymmetric anti-Hebbian learning function. **c** Symmetric Hebbian learning rule. **d** Symmetric anti-Hebbian learning function. Reprinted with permission from [62]

The fundamental formula to mathematically depict the STDP learning function is shown below [42]:

1 $$ \varDelta w=\xi \left(\varDelta T\right)=\left\{\begin{array}{l}{a}^{+}{e}^{-\Delta T/{\tau}^{+}\kern0.6em }\;\mathrm{if}\kern0.36em \Delta T>0\\ {}-{a}^{-}{e}^{\Delta T/{\tau}^{-}\kern0.24em }\mathrm{if}\kern0.36em \Delta T<0\end{array}\right. $$

where Δ*w* is the change on synapse weight, *ξ* denotes the STDP function, Δ*T* is the relative time difference between presynaptic spike (*t*_pre_) and postsynaptic spike (*t*_post_), and *a*_±_ and *τ*_±_ represent the scaling factor and the time constant of the exponential function, respectively. According to Eq. 1, four types of STDP updated functions commonly adopted by computational models of STDP synaptic learning can be readily derived [62], giving rise to Fig. 7. It should be noticed that the cases illustrated in Figs. 6 and 7a correspond to synapses with positive weights (*w* > 0). Therefore, as long as Δ*T* > 0, the synapse weight *w* is always strengthened due to the positive sign of Δ*w*. However, this learning rule does not apply to some so-called inhibitory synapses with negative weights (*w* < 0), as an increase in weight would in turn weaken the strength of the synapse. To resolve this issue, an STDP learning rule with a similar shape to Fig. 7b is required, since in this case decreasing the weight would strengthen the synapse itself when Δ*T* > 0. Such a relationship between Δ*w* and Δ*T*, given by Fig. 7b, is usually called anti-STDP learning function. Figure 7c, d is the symmetric form of (a) and (b), respectively.

STDP can be further categorized into additive STDP and multiplicative STDP. The learning function *ξ* of additive STDP is irrelevant to the actual weight (*w*), but strongly depending on the time interval (ΔT). Additive STDP requires the weight values to be bounded to an interval because weights will stabilize at one of their boundary values [63, 64]. On the other hand, the learning rule of multiplicative STDP relies on both actual synapse weight (*w*) and the time interval (Δ*T*). In multiplicative STDP, weight can stabilize to intermediate values inside the boundary definitions, whereby it is not mandatory to enforce boundary conditions for the weight values [63–65]. It should be kept in mind that STDP has recently attained much more interest than spike rate dependent plasticity (SRDP) due to its simplicity and biological plausibility as well as acceptable computational power.

**Fig. 8:**
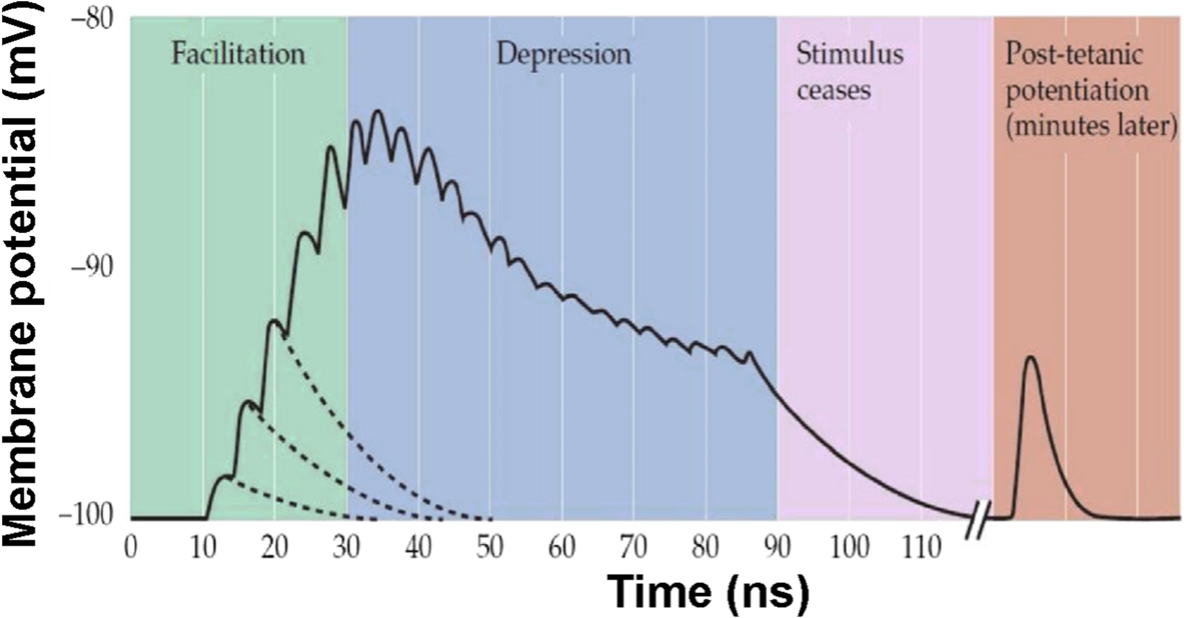
Experimental demonstration of STP for neuromuscular synapse. Presynaptic motor nerve was stimulated by train of electrical pulses. The end-plate potentials (EPPs) that are the depolarizations of skeletal muscle fibres caused by neurotransmitters binding to the postsynaptic membrane in the neuromuscular junction are facilitated initially, followed by a depression. Epps return to their rest state after removing the stimulation. Reprinted with permission from [5]

**Fig. 9:**
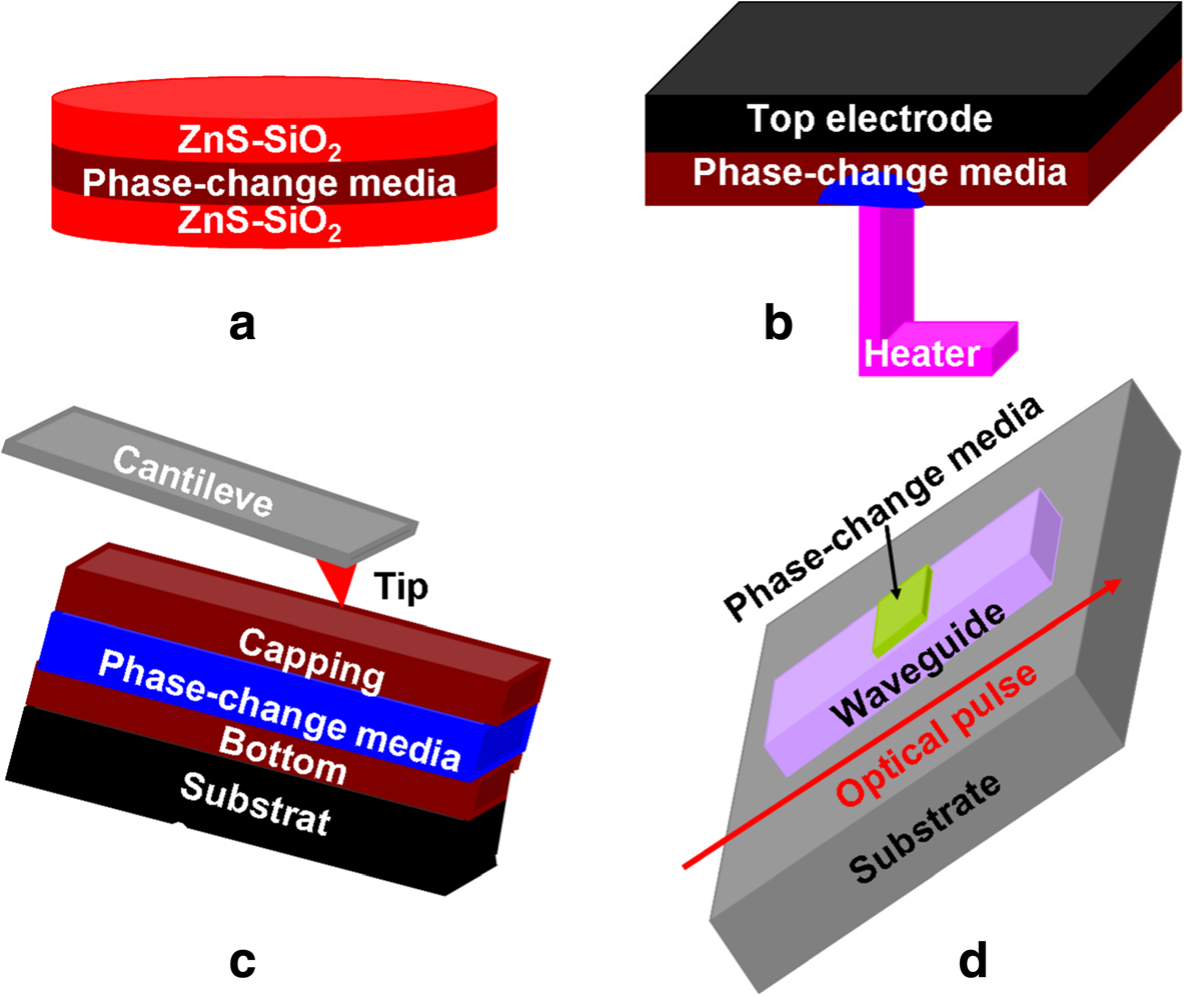
Application of phase-change materials on **a** phase-change optical disc, **b** PCRAM, **c** scanning probe phase-change memory, and **d** phase-change photonic memory

In contrast to long-term plasticity, short-term plasticity (STP) features the modification of synapse weight that can only last from tens of milliseconds to a few minutes and quickly recover to its initial state [66], as described in Fig. 9. Similar to long-term plasticity that can be classified into LTP and LTD, short-term plasticity also includes two types of activity patterns, which are short-term facilitation (STF) and short-term depression (STD). STF that transiently increases the synapse weight is primarily caused by the arriving of two or more action potentials at presynaptic neuron within a very short-time interval, thus boosting the concentration of Ca^2+^ ions. As a consequence, the subsequent presynaptic action potential can excite more neurotransmitter released, which can be further facilitated by a high-frequency train of presynaptic firings called tetanus. This leads to another form of synaptic plasticity referred to post-tetanic potentiation (Fig. 8). Repeated presynaptic activities can also cause continuous depletion of the synaptic vesicles available for release into the synaptic cleft, which results in decrease of synapse weight, i.e. STD.

### Phase-Change Materials

Undoubtedly building a neuromorphic circuit that can effectively imitate the behaviours and connections between different neurons is the most prospective approach to overcome von Neumann limits and to ultimately achieve neural brain emulation. However, conventional CMOS-based neuromorphic circuits caused much more energy consumption than the biological brain and cannot truly mimic its biological behaviours [67–70]. It is evident that a desired neuromorphic device must be able to emulate the spike-dependent synapse plasticity within the similar time interval to brain synapse while at the cost of low energy consumption. As a result, several innovative hardware architectures have been under intensive research aiming to achieve the required synapse performances. Within these technologies, PCM has been recently considered as a leading candidate in comparison with its rivals due to the analogous physical behaviours of phase-change materials to biological synapse [71, 72].

**Fig. 10:**
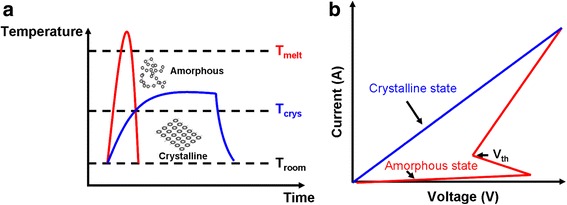
Schematic of **a** thermal switching and **b** electrical switching (i.e. threshold switching) in phase-change materials

Phase-change materials adopted for neuromorphic applications commonly refers to chalcogenide alloys that mainly include the elements Ge, Sb, and Te. Attractiveness of phase-change materials arises from a phenomenon that it can be switching reversibly between a metastable amorphous phase with high electrical resistivity and low optical reflectivity and a stable crystalline phase with low electrical resistivity and high optical reflectivity when suffering from either electrical or optical stimulus [73]. Such remarkable differences on the electrical/optical properties between amorphous and crystalline phases make phase-change materials very promising for a variety of NVM applications such as optical disc [33, 74], PCRAM [35, 75–77], scanning probe phase-change memory [36, 78–80], and phase-change photonic device [37, 81–83], as shown in Fig. 9. To electrically achieve crystallization, an electric pulse is applied to phase-change materials at amorphous state to bring its temperature above glass transition temperature but below melting temperature by means of the resulting Joule heating, subsequently followed by a slow cooling. This process usually refers to ‘SET’ operation for PCRAM operation. Amorphization of phase-change materials that is called ‘RESET’ by convention is induced by electrically heating the media above melting temperature and quickly quenching it to room temperature. In general, SET operation generally lasts a few hundreds of nanoseconds, while time taken by ‘RESET’ is in the range of a few of tens of nanoseconds or even down to picosecond regime. Such a thermal switching is schematically illustrated in Fig. 10a. In addition to thermal switching, phase-change materials also exhibit a unique electrical switching characteristic, namely ‘threshold switching’ [84–86]. Because of threshold switching effect depicted in Fig. 10b, the electrical resistance of the phase-change materials in amorphous state can be dramatically reduced once the bias voltage exceeds the threshold value, thereby resulting in a high current beneficial for the incoming crystallization. Threshold switching is extremely crucial for phase-change materials, as it allows for the phase transition at relatively low voltage. Otherwise, phase transition can only take place using very high voltage that yields unnecessary energy consumption. These superior physical properties consolidated with considerable practical and theoretical knowledge obtained from the applications of phase-change materials for NVM field during the last two decades successfully endow phase-change materials with several advantageous features such as an excellent scalability of <5 nm [87], a fast switching speed of <1 ns [88], a long cycle endurance of >10^12^ [17], and a low energy consumption of less than a petajoule per bit [89], thus enabling the viability of using nanoscale PCMs to emulate the biological synapse that has similar characteristics to phase-change materials.

### PCM-Based Synapse Models

Before the experimental study of the PCM-based artificial synapse, a comprehensively theoretical model is always preferable to assess the potential of PCM devices for large-scale spike neural network (SNN) applications. Without an effectively theoretical model, researchers may have to spend tremendous computational and experimental resources to establish the viable technological path towards the success of building PCM-based neurons and synapses. To achieve this goal, Suri et al. first proposed a behaviour model to simulate the LTP and LTD behaviours of PCMs using the Ge_2_Sb_2_Te_5_ (GST) and GeTe medium [90–92]. In Suri’s model, the conductances of GST and GeTe medium are considered as the synapse weight which can be therefore modified through either ‘SET’ process that corresponds to LTP or ‘RESET’ process indicating LTD. In this case, proper pulse magnitude and width need to be determined carefully so as to generate the desired conductances. The change on synapse weight (conductance in this case) with respect to the applied pulse duration is described as:

2 $$ \frac{dG}{dt}=\alpha \exp \left(-\beta \frac{G-{G}_{\min }}{G_{\max }-{G}_{\min }}\right) $$

where *G*, *G*_min_, and *G*_max_ denotes the device conductance, minimum device conductance, and maximum device conductance, respectively; *α* and *β* are fitting parameters.

According to above equation, the change on synapse weight during a certain time interval Δ*t* is equal to:

3 $$ d G=\alpha \varDelta t \exp \left(-\beta \frac{G-{G}_{\min }}{G_{\max }-{G}_{\min }}\right) $$

**Fig. 11:**
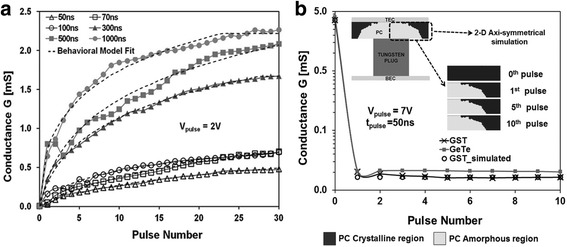
Theoretical fitting of the experimental **a** LTP and **b** LTD characteristics of the GST device using the developed behaviour model for different pulse numbers and width. Reprinted with permission from [90]

The reported equations here give rise to a satisfactory matching with the experimentally measured data for GST device, as shown in Fig. 11.

Although this model can accurately forecast the biological behaviours of brain synapses, it cannot be directly applied to a hybrid neural circuit comprising CMOS neurons and PCM-based synapse that requires a circuit-compatible model [90]. In order to resolve this issue, a more comprehensive circuit-compatible model consisting of electrical, thermal, and phase-change sections has been developed to mimic the progressive character of the LTP experiments [90]. Electrical sections is governed by the well-known Ohm’s law, and the adopted device resistance *R*_dev_ is the sum of the electrode resistance Rs and the resistance of the GST layer that depends on the volume fraction ratio of amorphous to crystalline phase as well as the resistance drift effect [93], given by

4 $$ {R}_{\mathrm{dev}}={R}_{\mathrm{s}}+{R_{\mathrm{c}}}^{\left(1-{C}_a\right)}+{R}_{0\mathrm{a}}{\left(\frac{t}{t_0}\right)}^{C_{\mathrm{a}} dr} $$

where *R*_c_ and *R*_0a_ is the resistance of fully crystalline and amorphous phase respectively and *C*_a_ is the amorphous volume fraction; *t*_0_ is the time when the initial phase-change process begins, and dr is an exponent that indicates the power-law slope. The thermal section calculates the temperature *T*_b_ inside the GST media using

5 $$ {T}_{\mathrm{b}}={T}_0+{P}_{\mathrm{t}}{R}_{\mathrm{t}\mathrm{gst}} $$

where *T*_0_ is the ambient temperature, *P*_t_ is the electrical power resulting from Joule heating, and *R*_tgst_ is the thermal resistance of the device, described by

6 $$ {P}_{\mathrm{t}}=\frac{u^2}{R_{\mathrm{s}}+{R}_{0\mathrm{c}}} $$

7 $$ {R}_{\mathrm{tgst}}=\left(1-{C}_{\mathrm{a}}\right){R}_{\mathrm{tc}0}+{C}_{\mathrm{a}}{R}_{\mathrm{ta}0} $$

where *u* is the applied electric pulse and *R*_tc0_ and *R*_ta0_ are the thermal resistance of the full crystallized and amorphized phases.

The phase-change section is mainly employed to calculate amorphous volume fraction Ca that strongly relates to the electrical and thermal sections using crystallization rate equation:

8 $$ \frac{d{ C}_{\mathrm{a}}}{ d t}=-\frac{{C_{\mathrm{a}}}^2}{\tau_{\mathrm{c}}}\left(1- \exp \left({E}_{\mathrm{a}}\frac{T_{\mathrm{b}}-{T}_{\mathrm{m}}}{T_{\mathrm{b}}}\right)\right) \exp \left(-\frac{E_{\mathrm{b}}}{T_{\mathrm{b}}}\right)\kern0.48em \mathrm{when}\kern0.24em {T}_{\mathrm{b}}>{T}_{\mathrm{m}} $$

9 $$ \frac{d{ C}_a}{ d t}=-\frac{1}{\tau_{\mathrm{a}}}\frac{T_{\mathrm{b}}-{T}_{\mathrm{m}}}{T_{\mathrm{m}}}\kern0.49em \mathrm{when}\ {T}_{\mathrm{m}}>{T}_{\mathrm{b}}>{T}_{\mathrm{g}} $$

where *T*_m_ and *T*_g_ are melting and glass transition temperature for GST; *E*_a_ and *E*_b_ are the fitting parameters for crystallization rate at high and low temperature, respectively; *τ*_a_ and *τ*_c_ are the fitting amorphization and crystallization rates, respectively.

**Fig. 12:**
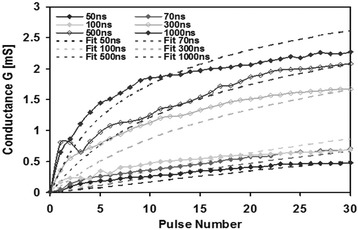
Theoretical fitting of the experimental LTP characteristics of the GST device using the developed circuit-compatible model for different pulse numbers and width. Reprinted with permission from [90]

By solving the aforementioned electrical, thermal, and phase-change sub-models simultaneously, this circuit-compatible model exhibits a less closer fitting to the observed LTP behaviour of the GST device in comparison with the previous behaviour model, as shown in Fig. 12. However, due to its capability of capturing the correct behaviour of PCMs for a relatively wide range of measurements with a small number of semi-physical parameters, it is therefore suitable for the easier circuit design that takes advantage of PCMs to emulate millions of synapses.

Another behaviour model to study the computational properties of networks of synapses based on phase-change materials was developed by Jackson et al. [94]. In this model, the state transition is determined by a calculated probability depending on the initial conductance and pre-post spike timing. In this case, the device conduction is extracted from a log-Gaussian distribution once the probability indicates the occurrence of the state transition. The pre-before-post pairings are tackled completely separately from post-before-pre pairings, each requiring a distinct set of model parameters. The probability of this phase-change-based synapse is given for each type of pairing between the amorphous and crystalline states as a function of the pre-post spike timing Δ*t*, tailored by the initial device resistance *R*_i_ following:

10 $$ P\left(\mathrm{transition}\right)={\left(1+ \exp \left(\frac{\varDelta t+\alpha { \log}_{10}{R}_i+\beta}{\kappa}\right)\right)}^{-1} $$

where *α*, *β*, and *γ* are timescale-dependent, threshold-dependent, and initial resistance-dependent parameters, respectively. Hence, the probability for a resistance *R*_*f*_ = 10^*x*^ is calculated by:

11 $$ P(x)=\frac{1}{\sqrt{2\pi {\sigma}^2}} \exp \left(-\frac{{\left( x-\mu \exp \left(\gamma \varDelta t\right)\right)}^2}{2{\sigma}^2}\right) $$

where *μ*, *σ*, and *γ* are parameters regulating the mean, standard deviation, and dependence on spike timing of the log normal distribution, respectively. It should be noticed that these parameter values rely on the physical characteristics of the device and the pulse scheme harnessed to induce phase transformations, showing a good agreement with the experimentally measured distribution of conductances and the STDP-like dynamics.

Differing from aforementioned models, the response of PCM-based synapse to the time delay between pre-post spikes has recently been re-examined by an ab initio molecular-dynamics (AIMD) model that consists of 180 atom models created in cubic supercells with periodic boundary conditions at a fixed density of 6110 kg/m^3^[95]. PAW pseudopotentials are deployed in this case to handle the outer *s* and *p* electrons as valence electrons [96], associated with the Perdew-Burke-Enzerhof (PBE) exchange-correlation functional and a plane-wave kinetic-energy cutoff of 175 eV [97]. During the simulation, phase-change materials, referring to GST, are initially considered to have amorphous phase obtained from a conventional melt-quench approach at a cooling rate of −15 K/ps. Crystallization in this model is achieve by heating it at 500 K for 500 ps, thus leading to the adequate number of the defined fourfold rings that represent the structural order of the GST media [98, 99]. According to this model, fourfold rings are interpreted as four atoms forming a closed path with a maximum bonding distance and with all four three-atom bond angles, plus the angle between the planes defined by two triplets of atoms, being a maximum of 20° from the ideal angles of 90° and 180°, respectively.

**Fig. 13:**
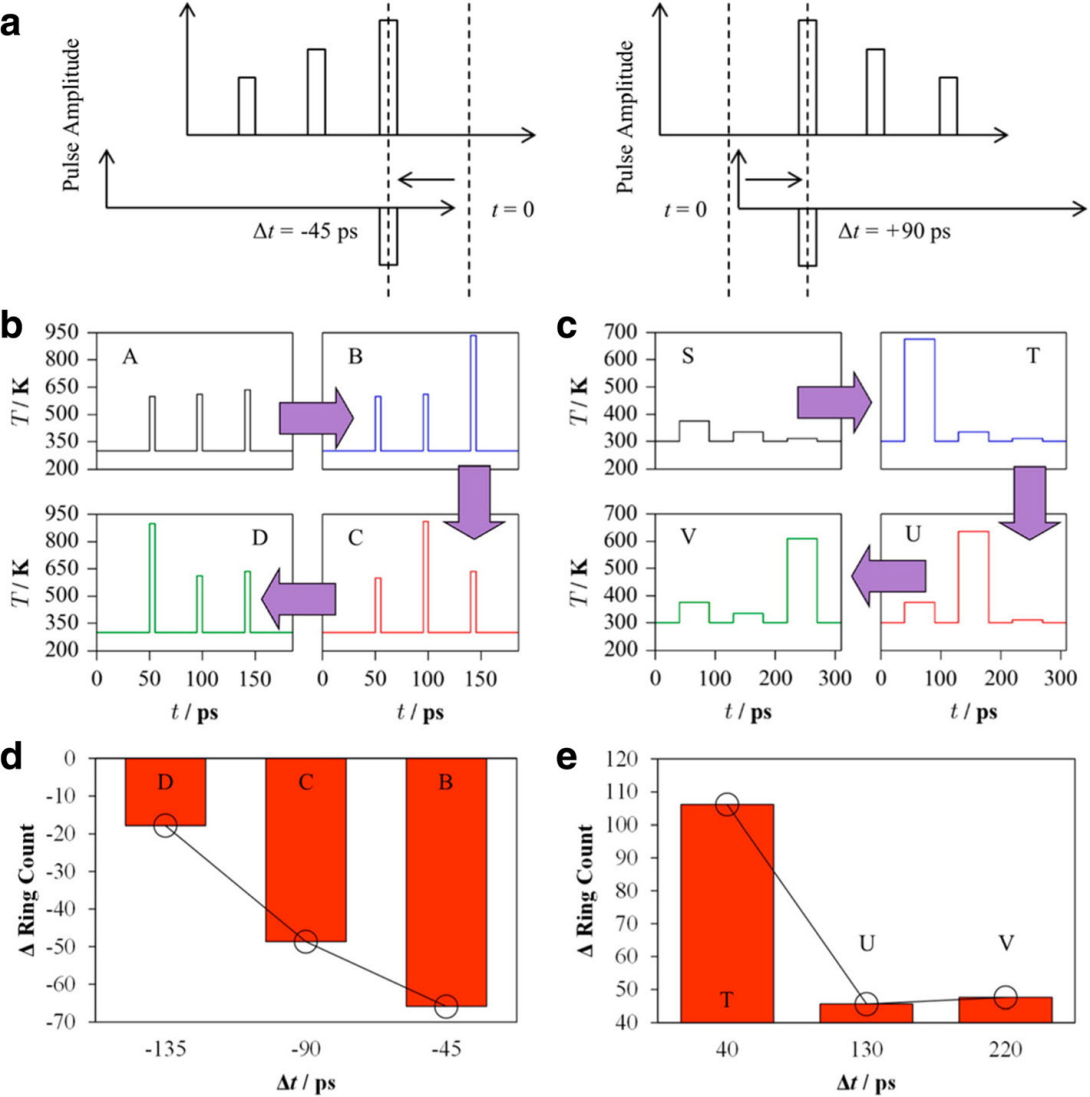
**a** The adopted pulse scheme used for the LTD simulation (*left*) and the LTP simulation (*right*). **b** Overlapping of pre-post spikes to simulate STDP behaviour of LTD. **c** Overlapping of pre-post spikes to simulate STDP behaviour of LTP. **d** Change in the number of fourfold rings at the end of the sequence for LTD simulation. **e** Change in the number of fourfold rings at the end of the sequence for LTP simulation. Reprinted with permission from [95]

**Fig. 14:**
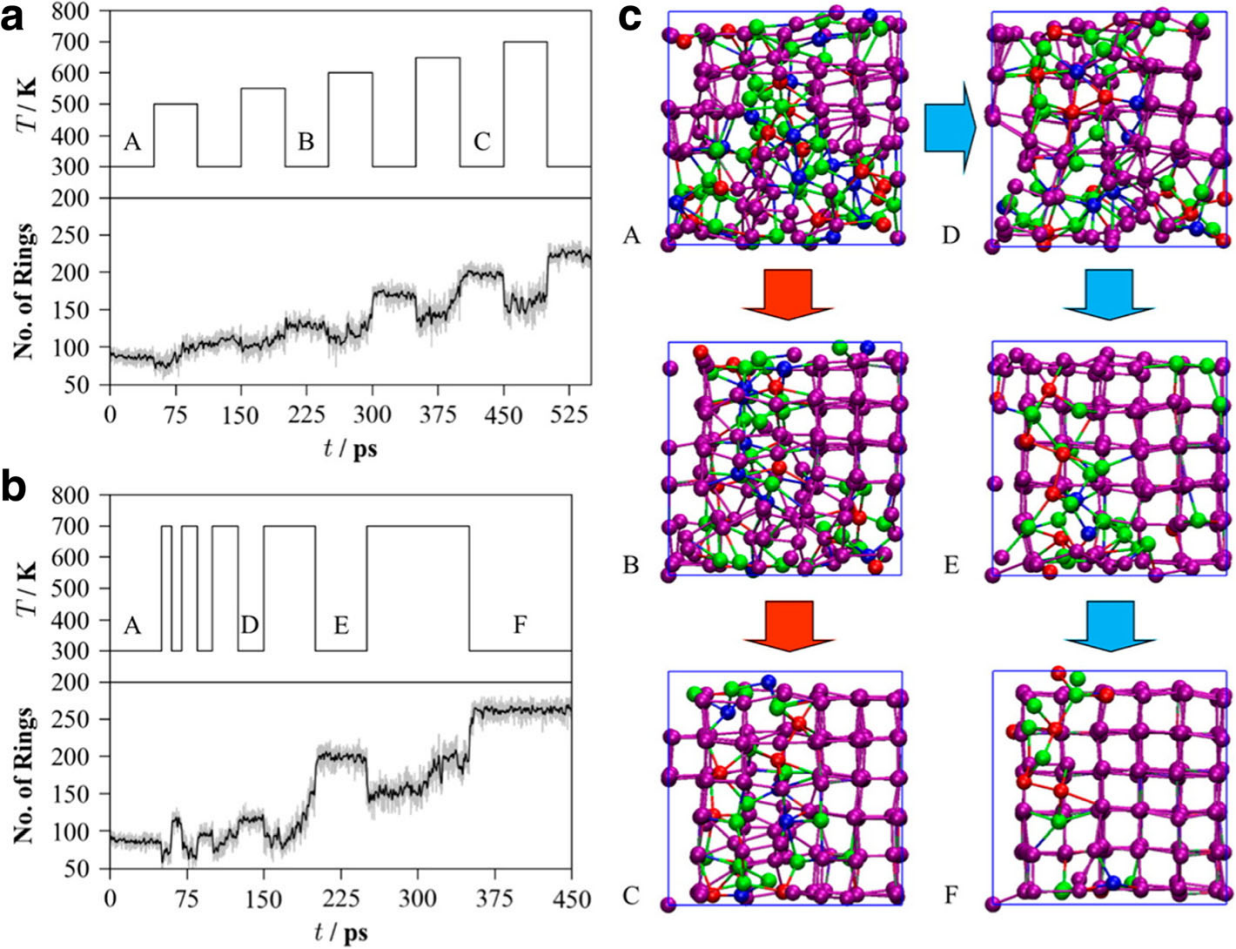
**a** Effect of a sequence of 50 ps heating pulses with gradually increased magnitudes (*top*) on the number of fourfold rings (*bottom*). **b** Effect of the temperature pulse duration with a constant magnitude of 700 K (*top*) on the number of fourfold rings (*bottom*). **c** The snapshots of the model are taken from the middle of the rest periods marked *A*, *B*, and *C* in (**a**) and *A*, *D*, *E*, and *F* in (**b**), and show the progressive growth of an initially small crystalline cluster. Atoms forming parts of fourfold rings are coloured *purple*, and the colour coding of the other atoms is as follows: Ge, *blue*; Sb, *red*; Te, *green*. Reprinted with permission from [95]

In order to simulate the STDP behaviour of the designed artificial synapse, a train of temperate pulses with different width and magnitudes are introduced into this model to represent the corresponding pre- and post-spikes. For LTD simulation, the pre-spikes comprise three stepcase 5-ps temperature pulses of 600, 610, and 635 K, separated by a time interval of 40 ps at 300 K between each single pulse. The pre-spikes are set to have sufficient magnitude but with too short duration to induce the crystallization. The post-spike consists of a sole temperature pulse of 300 K. As a result, by modulating relative time window between pre-post spikes, the single post-spike can overlap with the pre-spike with different temperature magnitude. As the superposed temperature exceeds the melting point, the resulting pre-post spikes would attenuate the degree of crystallinity of GST and thus decrease the conductivity, reflected in this model by a reduction in the number of fourfold rings. On the other hand, the pre-spikes for LTP simulation consist of a sequence of 50 ps temperature pulses of 375, 335, and 310 K with a time interval of 40 ps, while the post-spike remains the same as the LTD case. The choice for the LTP pre-spikes follows a rule that these temperature pulses have sufficiently long width but with too low amplitude to cause crystallization. Therefore, the resulting temperature possessed from the overlapping of pre-post spikes outnumbers the crystalline point and consequently boost a growth in structure order, increasing the conductivity. The described pulse scheme as well as the resulting influence on the structure order of GST is schematically shown in Fig. 13. Additionally, in order to deeply analyze and interpret the results shown in Fig. 13, the effects of various single pulse width and temperature on the characteristics of the designed PCM-based synapse are also evaluated, giving rise to Fig. 14. It is clearly indicated in Fig. 14c that a small stable crystalline cluster is formed by a low-temperature annealing of the melt-quenched amorphous model prior to the neuromorphic simulations, followed by a subsequent growth proportional to the magnitude and duration of the applied heat pulses, as illustrated in Fig. 13.

### PCM-Based Synapse Devices

**Fig. 15:**
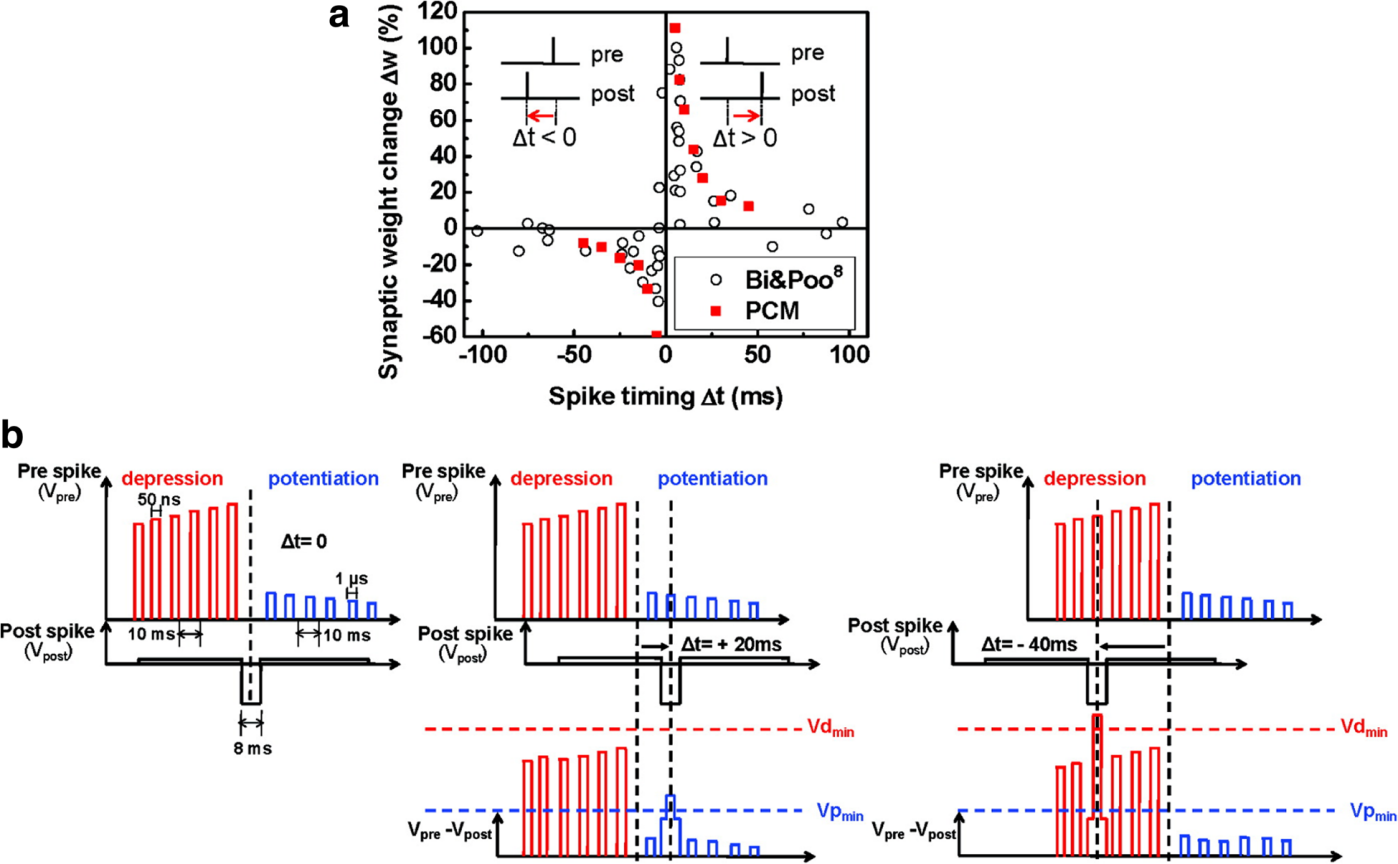
The adopted pulse scenario and the simulated STDP compared with the measured biological counterpart. **a** Measured STDP characteristic of the PCM-based synapse against the biological STDP measured from the hippocampal glutamatergic synapses. **b** The pulsing scenario to cause STDP for potentiation and depression processes. Reprinted with permission from [100]

The resulting theoretical feasibility secured from various in silico models has dramatically strengthened the confidence of worldwide researchers in physically realizing the desired PCM-based synapse. Thanks to this, the pioneering electronic synapse using phase-change materials was achieved by Kuzum et al. in 2011 to emulate biological STDP behaviour according to a very delicate control of the intermediate resistance level in the phase-change material (GST in this case) that is regarded as the synapse weight [100–102]. In their neuromorphic circuit, the pre-post spikes generated from an arbitrary waveform generator are connected to the top and bottom electrodes of an electronic synapse. Pre-spikes that consist of a train of stepwise pulses have successive larger magnitude for depression simulation corresponding to reset state, while exhibiting gradually decreasing magnitude for potentiation case indicated by set state. The pulse width and rise and fall times of a single depression pulse is chosen to be 50, 10, and 10 ns, whereas they are 1 μs, 100 ns, and 100 ns for a single potentiation pulse. The time interval between each depression or potentiaton pulse is 10 ms, and the entire duration of pre-spikes last 120 ms to match the biological synapse. In contrast to pre-spikes, post-spikes that operate as gating function for the pre-spike is a single pulse with a negative low magnitude and duration of 120 ms while having 8 ms at the centre. In this case, the net electric potential applied across the electronic synapse is equal to the difference between the magnitude of pre-spike and that of post-spike. If pre-post spikes are synchronous, the post-spike superposes with neither depression nor potentiation pulses of the pre-spikes. However, for the case of pre-spikes that precede post-spikes, the net potential drop across the designed synapse would exceed the so-called potentiation threshold, i.e. the minimum voltage to induce crystallization, which can increase the device conductance, suggesting an increase on synaptic weight. On the other hand, when the pre-spike lags behind the post-spike, the post-spike is overlapped with pre-spikes having larger magnitude so that the overall voltage drop would be above the depression threshold representing the minimum voltage for amorphization. This would effectively reduce the conductance, thus corresponding to a decrease on the synapse weight. More importantly, modulation of the relative time delay between pre-post spikes allows post-spike to overlap with pre-spike with different magnitudes and leads to various extents of either amorphous or crystalline states, closely mimicking the biological STDP mechanism. The presented pulsing scenario as well as the simulated STDP function is schematically shown in Fig. 15.

**Fig. 16:**
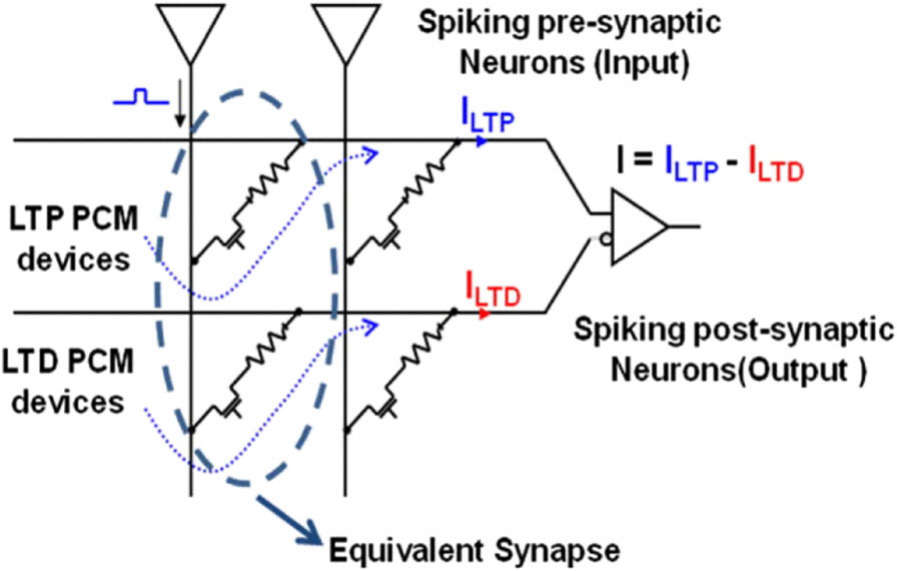
Circuit schematic for the 2-PCM synapse. The input of the current from the LTD devices is inverted in the post-synaptic neuron. Reprinted with permission from [90]

There is no doubt that the above approach can astonishingly imitate both LTD and LTP properties of biological synapse based on the adjustment of the intermediate resistance level. However, for implementation of large-scale neural systems, this complex resistance-control method would cause several severe issues such as capacitive line charging and high power dissipation [90]. In addition, as the emulation of the LTD process is strongly linked to amorphization, higher energy consumption is therefore required than the LTP process involving crystallization with less energy consumption. To address these issues, a novel paradigm to harness two PCM devices to simulate a single synapse, namely 2-PCM synapse, has been proposed by Suri et al. [90, 91], as illustrated in Fig. 16.

According to Fig. 16, an electronic synapse consists of one PCM device responsible for the LTP and another PCM device in charge of the LTD. The initial phases of both devices are considered to be amorphous. In contrast to the previous design, both LTP and LTD characteristics in this 2-PCM synapse are realized through the crystallization of phase-change materials. The LTP PCM device gives a positive current distribution, whereas current flowing through the LTD PCM device is negative by means of an inverter. Therefore, the potentiation of the LTD device in turn leads to a synaptic depression due to the subtracted current through it in the post-neuron. The pre-post spikes are configured in such a way that the overall impact of the two pulses only potentiate or partially crystallize the LTP device without affecting the LTD device in the case of pre-spike fired before post-spike, while the superposition of the two pulses only potentiates the LTD device rather than the LTP device when the post-spike precedes the pre-spike. Following above rule, the pre-neuron would send out a read pulse and enters ‘LTP’ mode for time *t*_LTP_ during which the LTP synapse undergoes a partial SET pulse if the postsynaptic neuron spikes; otherwise, the LTD synapse is programmed. One prominent advantage of this 2-PCM synapse obviously arises from its operations in crystalline regime for both LTP and LTD events that consumes less energy than amorphization case. Additionally, as the information stored in the designed device is chiefly in crystalline state, it is therefore less susceptible to the resistance drift effect that often occurs in phase-change materials in amorphous states.

**Fig. 17:**
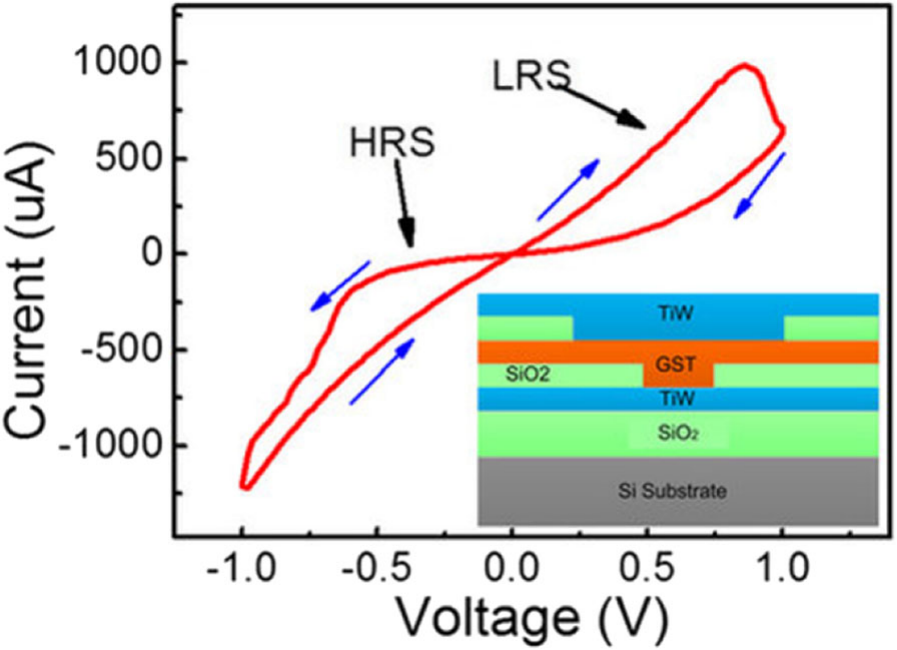
I–V characteristics measured by DC double sweeping, exhibiting a memristive hysteresis loop. Reprinted with permission from [103]

**Fig. 18:**
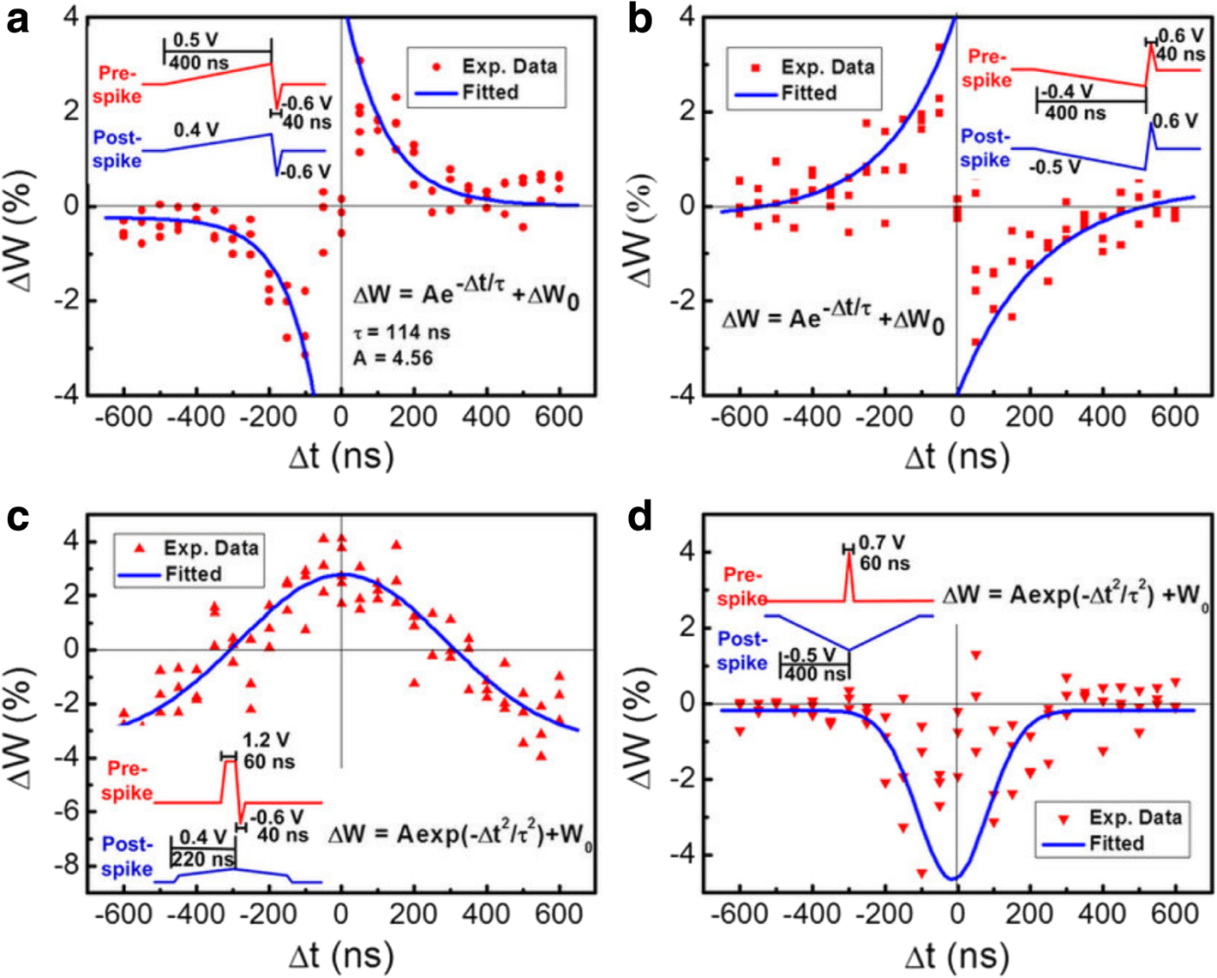
Four types of the STDP characteristics obtained from the c-GST-based electronic synapse. **a** Antisymmetric Hebbian learning rule. **b** Antisymmetric anti-Hebbian learning rule. **c** Symmetric Hebbian learning rule. **d** Symmetric anti-Hebbian learning rule. Reprinted with permission from [103]

Li et al. proposed another PCM-based synapse whose biological events are mainly controlled by crystallization rather than amorphization. In their experiment, the biological synapse is emulated by a crystalline GST (c-GST)-based memristor whose resistance varies between 500 Ω and 10 kΩ when subject to a DC voltage sweep [103, 104], as illustrated in Fig. 17. Accordingly, the synaptic weight is denoted by the conductance of the c-GST memristor that can be modulated by the applied electric pulses. It was found that the device conductance can be increased via the negative pulses that thus represent the potentiating spikes, while the positive spikes leading to the reduction on the device conductions corresponds to the depressing spikes. To trigger the synaptic behaviour in crystalline regime, the device was first crystallized with a resistance of 2.6 kΩ by a SET pulse. Similar to aforementioned pulse schemes, the potentiating spikes consist of a sequence of the pulse trains with successive increasing amplitude from −0.6 to −0.8 V with a 10-mV step, whereas the amplitude of the successive depressing spikes ranges from 1 to 1.8 V with a 40-mV step. The rest, rise, and trailing periods of a single spike is fixed to 30, 10, 10 ns, respectively. As a result, varying the relative time delay between pre-post spikes clearly results in various net voltage drops across the electronic synapse after the superposition of the pre- and post-spikes, thereby accurately resembling the typical or even more complex STDP forms, as shown in Fig. 18. Such an analogue phase-change synapse exhibits several advantages such as ultra-low operation voltage, ultra-fast synaptic events, and feasibility of time window tuning when compared to other emerging electronic synapses.

**Fig. 19:**
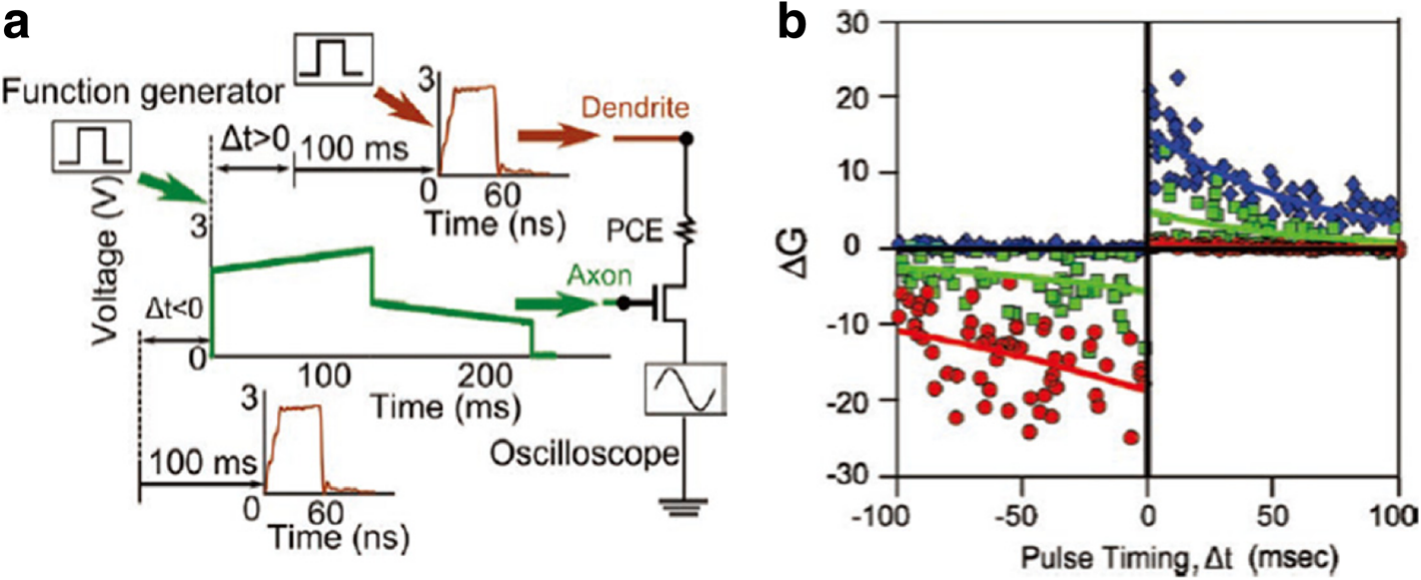
**a** The pulse algorithm adopted in [94] to emulate the STDP. **b** The generated STDP events using the designed circuit. Reprinted with permission from [94]

The practicality of using different pulsing schemes from above devices to generate STDP has recently been investigated by Jackson et al. who built an electronic synapse that includes a FET acting as an access device, connected in series with a PCM device [94]. A 200-ms electric pulse with front portion having higher amplitude and back portion having lower amplitude is injected to the gate of the synaptic FET with zero time delay after the presynaptic spike. Another 60-ns electric pulse with a time delay of 100 ms after postsynaptic spike is directly applied to the PCM device. The amplitudes of the pre-post spikes are established in such a way that the FET only programmes during the brief overlap between these two signals. According to this strategy, when the presynaptic neuron is spiked before post-neuron, the shorter pulse only coincides with the portion of the longer pulse with lower amplitude, whereby the resulting power only enables the crystallization and thus increases the device conductance. On the contrary, for the case of post-spike preceding the pre-spike, the overlapping of the shorter pulse with the portion of the longer pulse with larger amplitude would yield sufficiently high power to drive the temperature inside the phase-change materials towards the melting point, thus inducing amorphization and decreasing the conductance. Therefore, the consequent device conductance entirely depends on the coincidence of the short and long electrical signals whose characteristics can be modulated by the relative pulse time window. Such a modulation method in associated with the simulated STDP is given in Fig. 19.

**Fig. 20:**
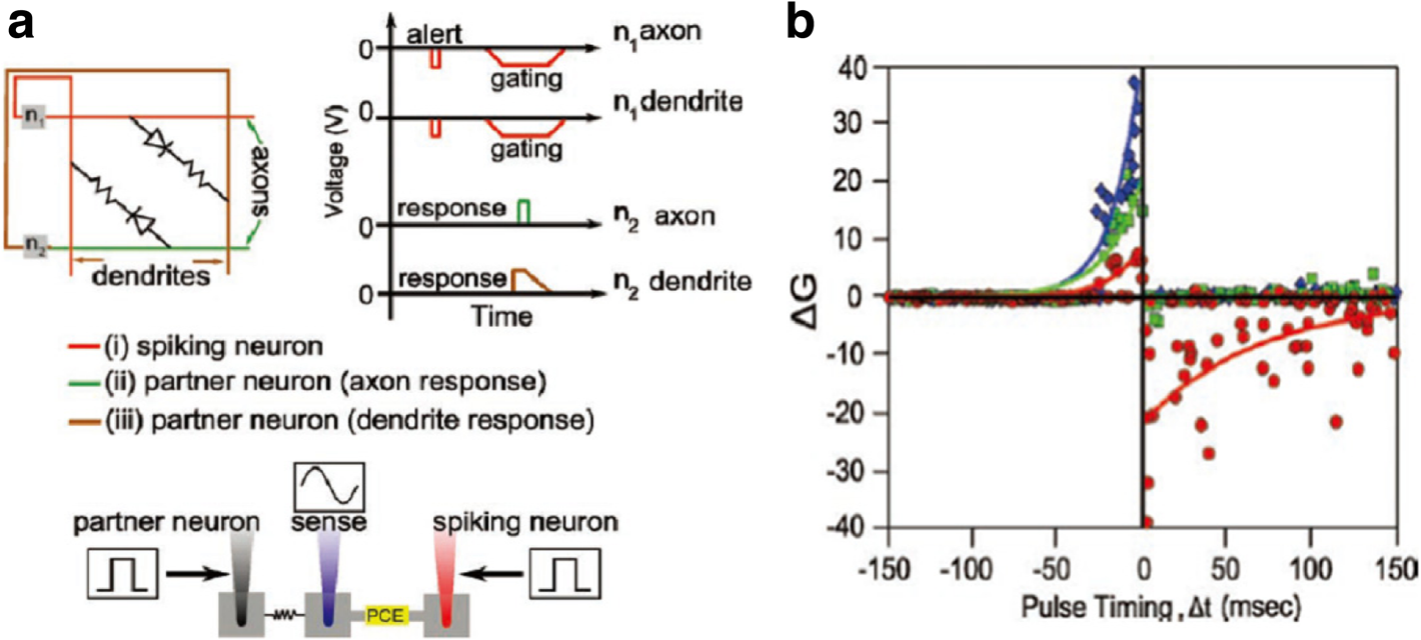
**a** The RC delay algorithm adopted in [94] to emulate the STDP. **b** The generated anti-STDP events using the designed circuit. Reprinted with permission from [94]

Differing from the first route, Jackson et al. also devised another electronic device to produce STDP where time delay is internally determined by a simple RC circuit [94], as revealed in Fig. 20. The memory of neuron’s latest firing event in this method is interpreted by the spike-timing-dependent change in synaptic conductance determined by an asynchronous handshaking mechanism. The key insight of this mechanism is that the spiking neuron would initialize its capacitor as well as fire a short ‘alert’ pulse to its synaptic partners. The function of the alert pulse is to remind the synaptic partners to examine the instantaneous voltages across their respective capacitors and to stimulate a response pulse after a constant delay that is therefore synchronous with a gating pulse from the spiking neuron. Therefore, the device can only be programmed during the overlapping of the gating and response pulses, which implies that only the synapses in conjunction with the spiking neuron can be programmed for large crossbar array. The width of the gating pulse offsets any unwanted temporal jitter between the arrival of the alert and response pulses; the amplitude change of the synaptic weight relies on the amplitude of the response pulse whose shape is determined by whether the response pulse is sent to an axon or a dendrite. Note that axon usually generates a square response pulse with very short falling time, thus giving rise to a very high quenching rate and decreasing the device conductance due to the amorphization. By contrast, response pulses obtained from dendrites are triangular shaped, which would increase the device conductance because of the crystallization effect. Based on this algorithm, the exponential time dependence of RC circuits permits the neurons that have not fired recently to only send low magnitude response signals. The merit of this newly proposed method arises from the fact that the neuronal spiking duration in this case is a sum of nanosecond-scale alert, nanosecond to microsecond delay, and nanosecond to microsecond gating pulses, resulting in a total duration on the order of a few microsecond that exceeds the biological synaptic rate at the same delay level of 100 ms.

### PCM-Based Neural Networks

**Fig. 21:**
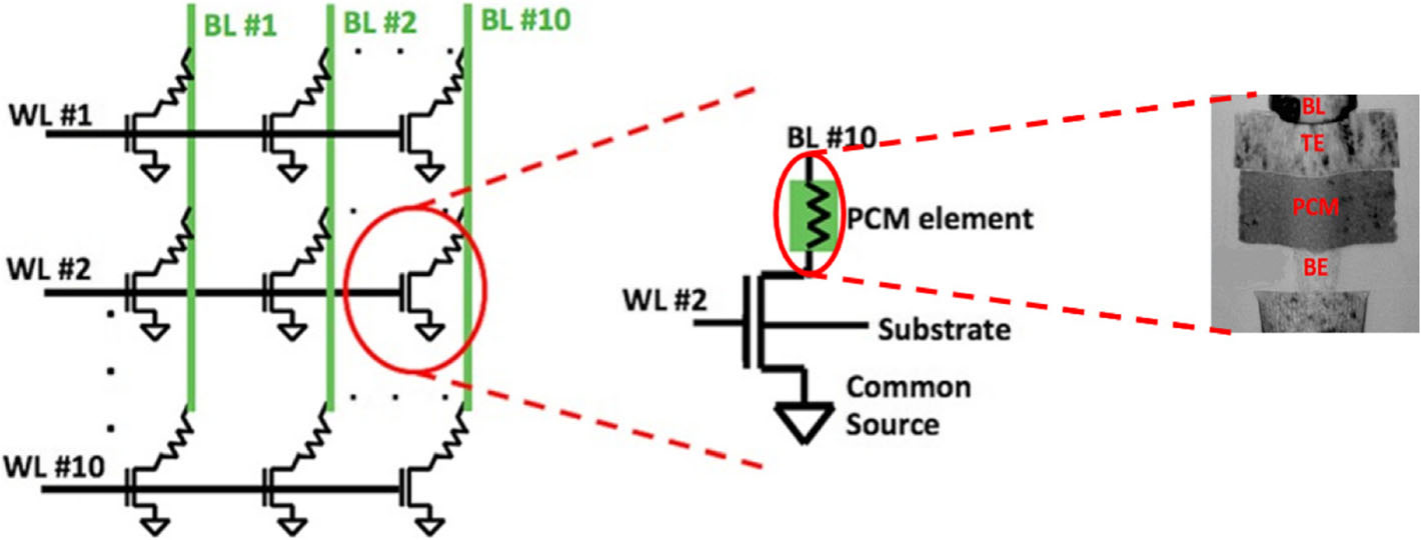
Schematics of a 10 × 10 PCM cell array (*left*), a single PCM cell (*middle*), and a mushroom type PCRAM (*right*). Note that the PCRAM cell adopted in [105] consists of a bottom electrode (BE), a heater, a phase-change layer, and a top electrode (TE). Reprinted with permission from [105]

Considering the large amount of biological synapses in human brain, it would be imperative to design a much more complex circuit than a single PCM-based synapse in order to emulate the STDP events at the network level. To achieve this goal, the idea to make use of PCM cells in a crossbar fashion to mimic the brain neural networks has been experimentally demonstrated by Eryilmaz et al. As can be seen from Fig. 21, a 10-by-10 memory array consisting of 100 PCM cells configured in a crossbar fashion are deployed in their experiment to imitate the large networks [105]. Each cell comprises a PCM device using a conventional mushroom type PCRAM and a selection transistor. Each cell can be accessed either through a bitline (BL) that is connected to the gates of selection transistors of 10 memory cells, or a wordline connected to the top electrode of the PCM element of 10 memory cells. According to this design, biasing the corresponding BL and WL nodes allows each cell to be exclusively accessed. To simulate the synaptic events, a pulse scheme that harnesses a RESET pulse of 1.1 V, followed by nine SET pulses of 0.85 V, was implemented to induce the gradual resistance change between high resistance and low resistance states, thus leading to nine distinguishable resistance levels.

**Fig. 22:**
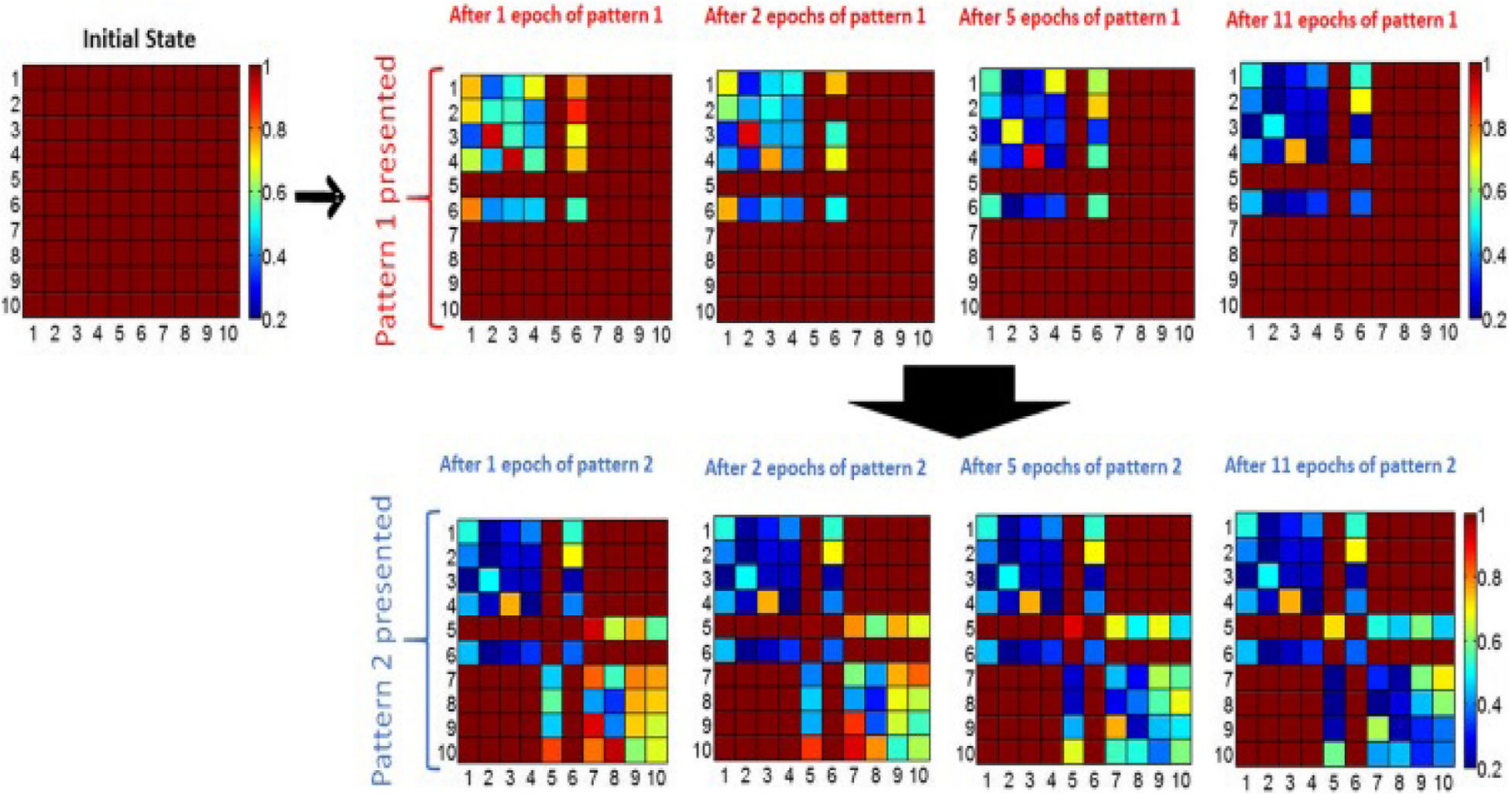
Evolution of normalized resistance of synaptic devices is shown, for the 60% initial variation case. All normalized resistances are one initially since the normalized resistance map shows the current resistance of a synaptic device divided by its initial resistance. Note that the row and column numbers corresponds to BL and WL that connect the synaptic devices. For instance, the data shown in row #3 and column #6 is the normalized resistance of the memory cell that can be accessed by BL #6 and WL #3. First, pattern 1 is presented to the network. For pattern 1, ON neurons for the complete pattern during update phase are N1, N2, N3, N4, and N6, and for the recall phase, N6 is OFF and expected to be recalled (i.e. expected to fire) after training with a certain number of epochs. The gradual decrease in the normalized resistance of synaptic connections between firing neurons during the update phase can be observed. After 11 epochs, when recall phase is performed, OFF pixel #6 (neuron #6) is recalled (meaning neuron #6 fires in recall phase), and then pattern 2 is presented for training. For pattern 2, the complete pattern is represented by N5, N7, N8, N9, and N10, and N5 is missing in the recall phase. Reprinted with permission from [105]

A more attractive feature of the designed phase-change synapse array stems from its ability to perform the associative learning function. Before the experiment, all the synapses are initially set to amorphous state, and the learning experiment consists of epochs during which synaptic weights are updated depending on firing neurons. The pattern is monitored after the training but with an incorrect pixel, and the incorrect pixel is expected to be recalled in the recall phase after training is performed. Therefore, a complete pattern appears during the training phase of an epoch, while an incomplete pattern with an incorrectly OFF pixel is presented during the recall phase. All patterns consist of 10 pixels, and each neuron is associated with a pixel. By means of this paradigm, test pattern was found to be stored and recalled associatively via Hebbian plasticity in a manner similar to the biological brain, as verified in Fig. 22.

**Fig. 23:**
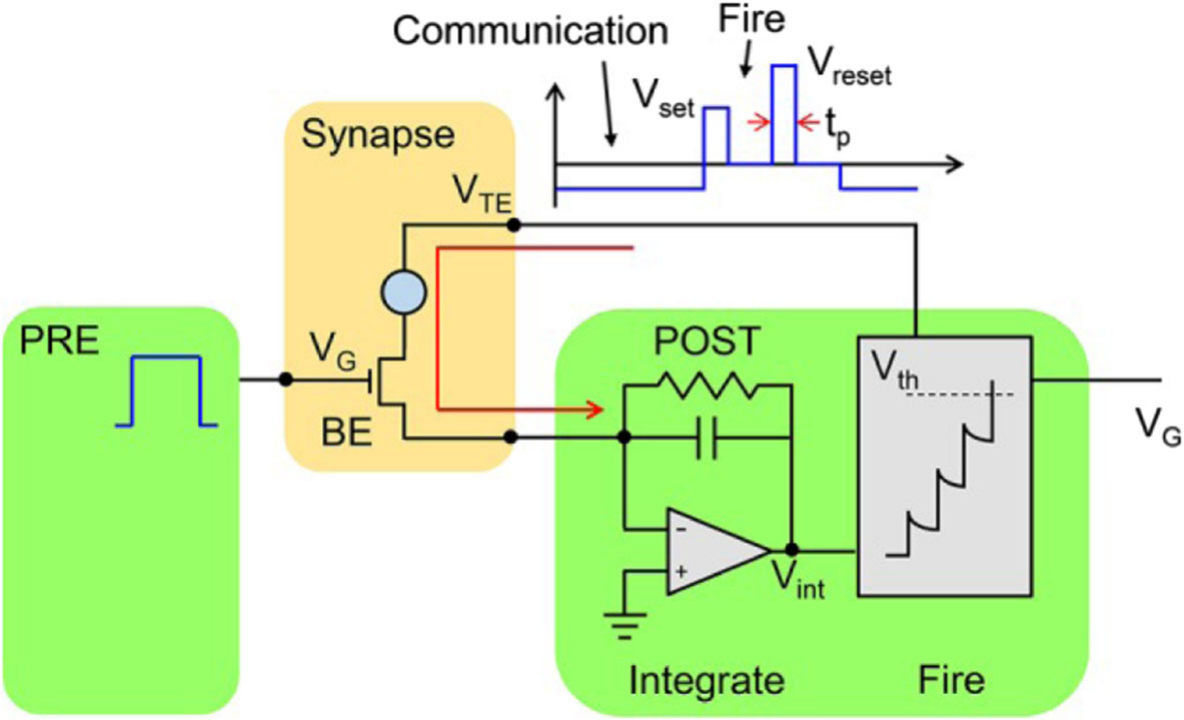
Schematic of the 1T1R synapse. Reprinted with permission from [106]

**Fig. 24:**
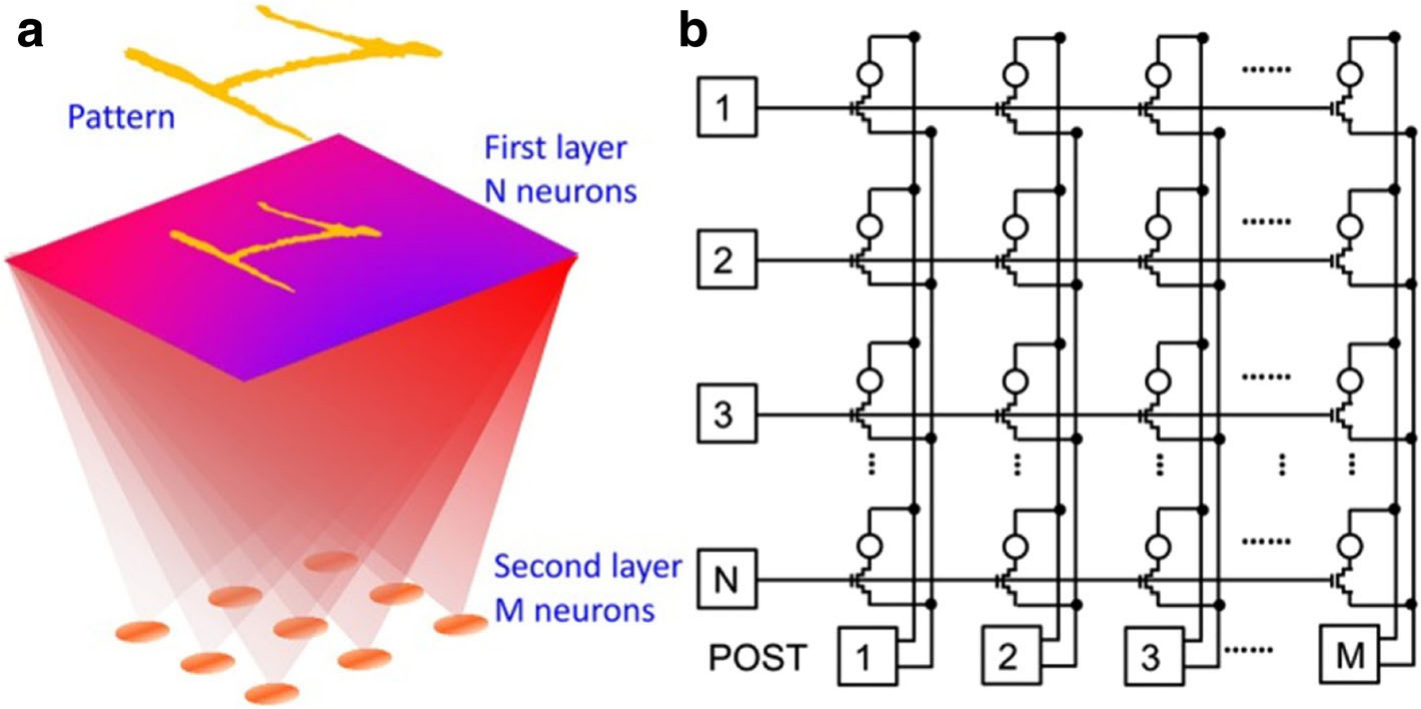
Schematic illustration of **a** two-layer neuron structure consisting of **b** a 1T1R synapse array. Reprinted with permission from [106]

Most recently, Ambrogio et al. has proposed another physics-based model to simulate the large-scaled synaptic networks that consist of numerous phase-change synapses with one transitor/one resistor (i.e. 1T1R) architecture [106, 107], as illustrated in Fig. 23. It is clearly indicated in Fig. 23 that the pre-spike defined as a train of rectangular pulses is directly applied to the gate of the transistor, and the positive gate voltage consolidated with a constant bias maintained by the postsynaptic neuron (POST) at *V*_TE_ triggers a current spike in the synapse that flows towards POST. As *V*_TE_ is usually set to be negatively constant, the resulting current spike is also negative and thus yields a staircase-like increase of the internal potential *V*_int_ in the inverting integrator. Once *V*_int_ exceeds the threshold voltage of the comparator *V*_th_, the POST excites a forward spike to the neuron in the next layer and a backward spike to *V*_TE_ to induce STDP through the change on the synaptic weight (PCM conductance here). *V*_TE_ spike in this case is made up of two rectangular pulses; the second of which is required to have higher amplitude than the first one. Therefore, the adopted shape of the VTE spike can effectively modulate the conductance of PCM device as a function of the relative time delay between pre-post spikes, clearly fitting the STDP behaviours. Furthermore, in order to prove its learning capability, simulations of pattern learning on a two-layer networks consisting of 28 × 28 pre- and one post-neuron with the proposed 1T1R synapse was performed, resulting in Fig. 24. The developed model allows for MNIST digit recognition probability of 33% and a corresponding error of 6%, which can be further improved to 95.5 and 0.35% respectively by using three-layer structure with 256 neurons [106].

### PCM-Based Neurons

No doubt that all results presented so far are dedicated to the emulation of biological synapse using PCM device. However, in order to ultimately achieve the brain-like neural networks, the capability of using PCM-based device to imitate biological neuron with the involvement of maintenance of the equilibrium potential, the transient dynamics, and the neurotransmission process is highly preferred. The prerequisite for PCM-based neuron realization is to effectively simplify the complex biological neuron mechanism that can be therefore readily applied to hardware [108]. Unlike the PCM-based synapse with continuously adjustable device conductance, the PCM-based neuron must be able to ‘fire’ after receiving a certain number of pulses that can influence an internal state that does not necessarily relate to the external conductance unless the neuron fires when exceeding the threshold. Additionally, non-volatility usually required for electronic synapse is not mandatory for neuron emulation that can utilize volatility to implement leaky integrate-and-fire dynamics.

**Fig. 25:**
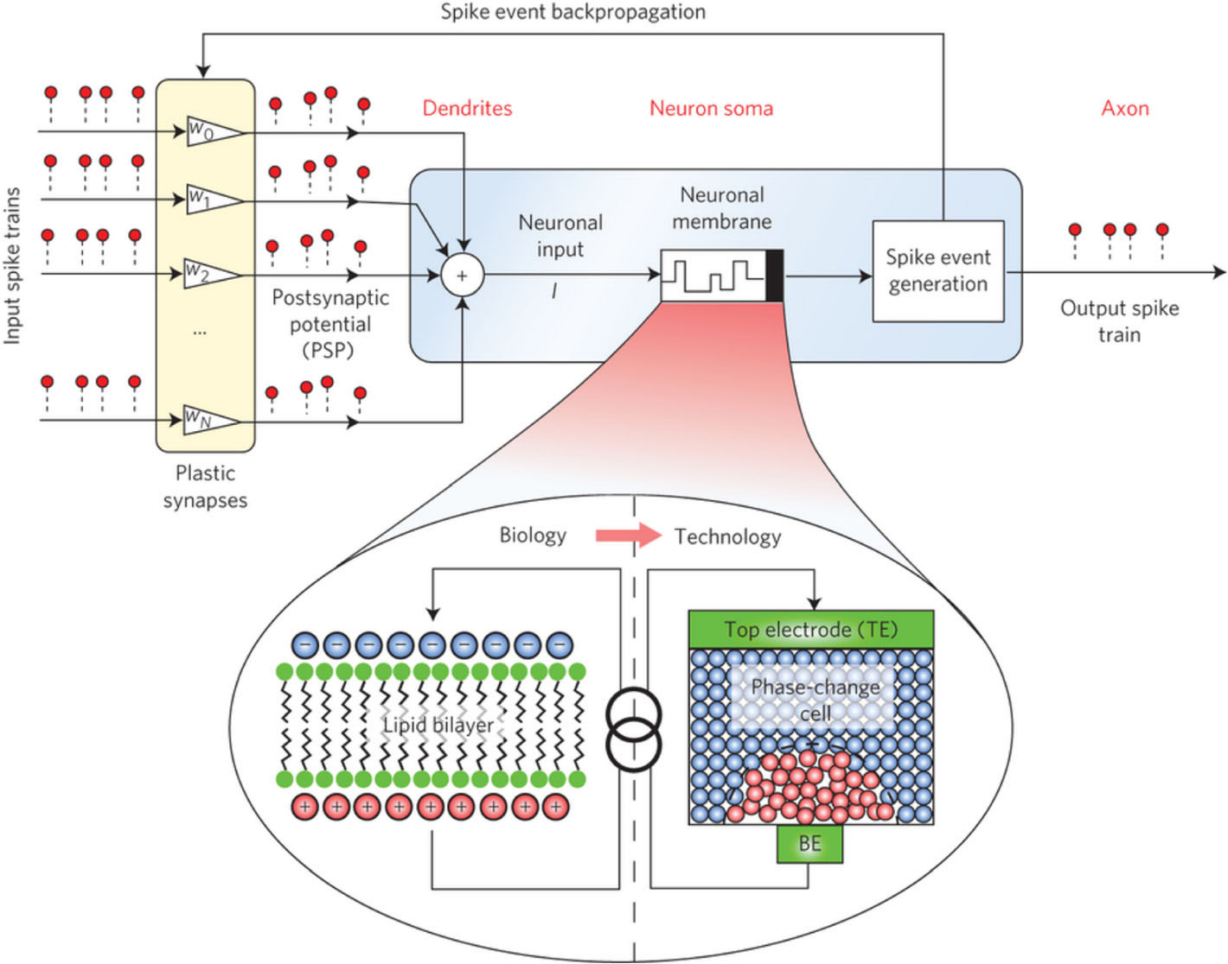
Schematic of PCM-based neuron with an array of plastic synapse at its input. Reprinted with permission from [110]

When simulating the biological neuron, particular attentions need to be paid to stochastic neuronal dynamics that was reported to account for signal encoding and transmission besides the deterministic neuronal dynamics [3]. The cause of this stochastic behaviour can be owed to several complex phenomena such as inter-neuron morphologic variabilities, chaotic motion of charge carriers due to thermal noise, ionic conductance noise, and other background noise [109]. As a result, it would be necessary for artificial neuron to reflect this stochastic firing behaviour so as to closely mimic the biological brain. In spite of these stringent requirements, Tuma et al. have recently devised a PCM-based neuron shown in Fig. 25 to integrate postsynaptic inputs [110], whereby the evolution of neuronal membrane potential as encoded by phase configuration within the device was demonstrated. More importantly, the ability to present remarkable inter-neuronal and intra-neuronal randomness using the devised neuron was also verified. Intra-device stochasticity that is usually ascribed to shot-to-shot variability in both internal atomic configuration of the melt-quenched amorphous region and thickness results in multiple integrate-and-fire cycles in a single phase-change neuron to generate a distribution of the interspike intervals, leading to population-based computation. Notwithstanding the slow firing rate of the individual neurons, overall neuron population was proved to accurately indicate fast signals. Based on the designed PCM-based neuron, the detection of temporal correlations within a large number of event-based data streams was demonstrated, and a complete PCM neuromorphic circuit made up of PCM-based neurons and PCM-based synapses has also been delivered [111].

## Conclusions

The ability to gradually induce a reversible switch between SET and RESET states, integrated with several superior transition properties such as fast switching speed, low energy consumption, and long retention, has made phase-change-based devices a leading candidate to emulate the biological synaptic events. Moreover, the excellent scalability of phase-change materials down to 2-nm size [112] also forecasts its potential to reproduce the ultra-high density neurons and synapses inside human brain. In this case, majority of the current work on electronic synapse are devoted to simulate the STDP event of the biological synapse that was reported to govern the learning and memory function by gradually changing the conductance of the phase-change materials, thereby resulting in several novel pulse schemes to adjust the device conductance with respect to the relative time delay between two external stimulus applied to device that represent pre-post spikes. Most importantly, the construction of PCM elements in array level allows for the emulation of large-scale connectivity of human brain with any given neuron having as many as 10,000 inputs from other neurons, which is exemplified by an integrated hardware with 256 × 256 neurons and 64,000 synapses [113].

**Fig. 26:**
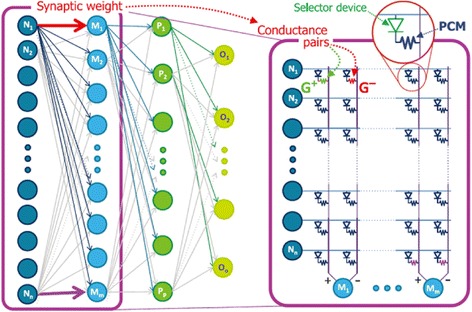
Implementation of dense crossbar of PCM and selector devices for non-Von Neumann computing where neuron activates each other through dense networks of programmable synaptic weights. Reprinted with permission from [114]

In spite of the aforementioned merits, the neurons and synapses based on PCM devices are also facing some serious issues. Although the conductance of PCM device in crystalline phase can be modulated continuously, the device conductance in amorphous phase is found to suffer from a sudden change. This can be alleviated using multiple conductances per synapse and periodic corrections [3], but still remaining questionable. Besides, the inherent weakness of amorphous phase-change materials including resistance drift or relaxation of the amorphous phase after the melt-quenching also exacerbates the application of PCM on neuromorphic circuit systems. Another issue of PCM-based neuromorphic device arises from its fairly poor performance metrics mainly due to immaturities or inefficiencies in currently developed STDP learning algorithm. Under this circumstance, such inherent imperfections of the PCM devices may pose some indiscernible problems. To boost the classification accuracy, a so-called back propagation method that has received extensive application in computer science field to train artificial neural networks has recently been introduced a three-layer perceptron network with 164,885 synapses based on 2-PCM structure that was trained on a subset of a database of handwritten digits, leading to training and test accuracies of 82–83% [114], as shown in Fig. 26. This work simply implies that classification accuracy can be achieved on the condition that either phase-change materials or the training algorithm permits PCM devices to serve more like a bidirectional NVM with a symmetric, linear conductance response of high dynamic range [115].

Despite the aforementioned challenges, the excellent physical properties of phase-change materials in conjunction with the currently mature technologies on PCM devices has provided an opportunity to envision the success of the future artificial neural networks that can perform the similar complex tasks to human brain while without sacrificing the occupied area and energy consumption. Device models suitable for neuromorphic architectures are still needed for application-specific performance evaluations of these systems.
